# Discovery of VU6077564: A Selective M_1_ Antagonist That Promotes Neurite Outgrowth in Adult Sensory Neurons

**DOI:** 10.1021/acschemneuro.6c00367

**Published:** 2026-07-07

**Authors:** Tomayo I. Berida, Cayden J. Dodd, Joshua C. Wilkinson, Darrell R. Smith, Sichen Chang, Christopher C. Presley, Li Peng, Snehal Sant, Srinivasan Krishnan, Irene Zagol-Ikapitte, Katherine J. Watson, Analisa Thompson Gray, Alice L. Rodriguez, Hyekyung P. Cho, Carrie K. Jones, Olivier Boutaud, Colleen M. Niswender, Darren W. Engers, Paul Fernyhough, Craig W. Lindsley, Elizabeth S. Childress

**Affiliations:** † Warren Center for Neuroscience Drug Discovery, 5718 Vanderbilt University, Nashville, Tennessee 37232, United States; ‡ Department of Pharmacology, 12327 Vanderbilt University School of Medicine, Nashville, Tennessee 37232, United States; § Vanderbilt Institute for Therapeutic Advances, Vanderbilt University, Nashville, Tennessee 37232, United States; ∥ Division of Neurodegenerative & Neurodevelopmental Disorders, St. Boniface Hospital Albrechtsen Research Centre, 8664 University of Manitoba, Winnipeg R3T 2N2, Canada; ⊥ Vanderbilt Brain Institute, Vanderbilt University School of Medicine, Nashville, Tennessee 37232, United States; # Vanderbilt Kennedy Center, Vanderbilt University School of Medicine, Nashville, Tennessee 37232, United States; ∇ Department of Pharmacology & Therapeutics, Max Rady College of Medicine, Rady Faculty of Health Sciences, University of Manitoba, Winnipeg R3E 0T6, Canada; ○ Department of Chemistry, Vanderbilt University, Nashville, Tennessee 37232, United States; ◆ Department of Biochemistry, Vanderbilt University, Nashville, Tennessee 37232, United States

**Keywords:** Muscarinic acetylcholine receptor (mAChR), M_1_, antagonist, peripheral, neuropathy, neurite outgrowth, pharmacokinetics, VU6077564

## Abstract

Previously, we reported the potent and highly selective M_1_ antagonists **VU0415248** and **VU0452865**; however, these compounds were limited by high predicted hepatic clearance in rat. This work reports the development of cyclobutylsulfonamide-based analogs of **VU0415248** and **VU0452865**. From this exercise, we identified **VU6077564**, a peripherally restricted and selective M_1_ antagonist. Furthermore, we demonstrate the ability of **VU6077564** to promote neurite outgrowth in adult rat dorsal root ganglia sensory neurons, highlighting the potential of selective M_1_ antagonists for the treatment of peripheral neuropathy.

## Graphical abstract

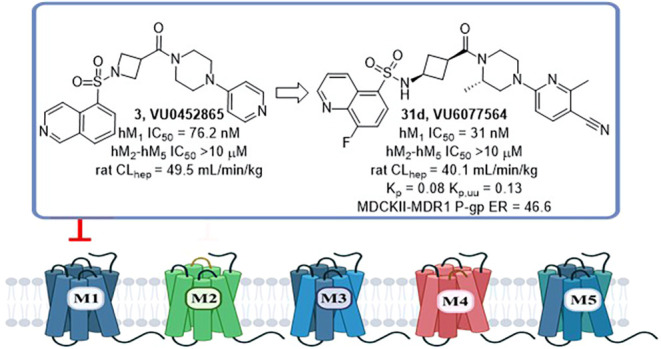

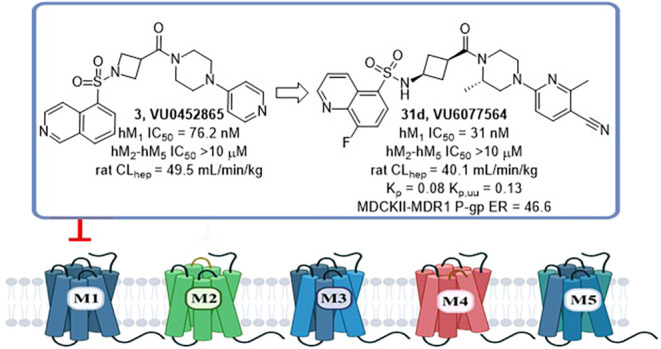

## Introduction

Acetylcholine (ACh), the primary neurotransmitter and neuromodulator of the cholinergic system, exerts its effects through two major classes of receptors: muscarinic acetylcholine receptors (mAChRs) and nicotinic acetylcholine receptors (nAChRs). These receptors are distributed throughout both the central nervous system (CNS) and the peripheral nervous system (PNS), where they regulate essential functions such as memory, learning, attention, arousal, and involuntary muscle movement. The muscarinic receptors are a family of five (M_1_–M_5_) G protein-coupled receptors (GPCRs) belonging to the α-branch of class A. The M_1_, M_3_, and M_5_ subtypes preferentially couple to G_q/11_, resulting in stimulation of phospholipase C (PLC) and intracellular calcium mobilization.^1^ The M_2_ and M_4_ subtypes primarily couple to G_i/o_ proteins, which primarily inhibit cAMP production from ATP by inhibiting adenylate cyclase (AC).^2^

Of the mAChRs, M_1_ accounts for 60% of mAChRs expressed in the CNS, primarily in the cerebral cortex, hippocampus, and striatum, emphasizing its vital role in synaptic plasticity, learning, and memory.^1,3^ Antagonism of M_1_ has shown potential for the treatment of conditions and diseases such as multiple sclerosis, Parkinson’s disease, and Fragile X syndrome.^4–6^ Beyond the CNS, M_1_ has also emerged as a therapeutic target for maladies of the PNS. We have previously demonstrated that M_1_ antagonists enhance neurite outgrowth and may represent potential therapeutic agents for peripheral neuropathy by promoting axonal repair across a variety of neuropathic animal models.^7^ Notably, in cultured adult dorsal root ganglia (DRG) sensory neurons the pan-muscarinic antagonists hexamethonium and mecamylamine did not exert any detectable effect in elevating neurite outgrowth. However, M_1_ selective antagonists pirenzepine, muscarinic toxin 7 (MT7), and ML012 (**VU0255035**) (Figure 1) demonstrated significant activation of AMP-activated protein kinase (AMPK) and augmentation of mitochondrial function to drive elevated neurite outgrowth.^7^ The translational significance of M_1_ antagonists was recently highlighted in phase 2 clinical trials in patients with type 2 diabetes and exhibiting mild-moderate sensory neuropathy. For 5–6 months, daily topical treatment with oxybutynin (a potent, nonselective M_1_ antagonist that is available over-the-counter) or pirenzepine (a selective M_1_ antagonist with limited penetration into the CNS) elevated nerve fiber levels in the skin as well as reversed clinical signs of pain and improved quality of life (as assessed using the Norfolk scale).^8,9^ Future development of oxybutynin for therapy in diseases of the PNS is discouraged due to its lack of selectivity for M_1_ and propensity to cross the blood-brain barrier.^10^ Consequently, M_1_ antagonists with improved selectivity and enhanced PNS distribution compared with the CNS are needed to further investigate the therapeutic potential of M_1_ blockade in treating peripheral neuropathy.

**Figure 1:**
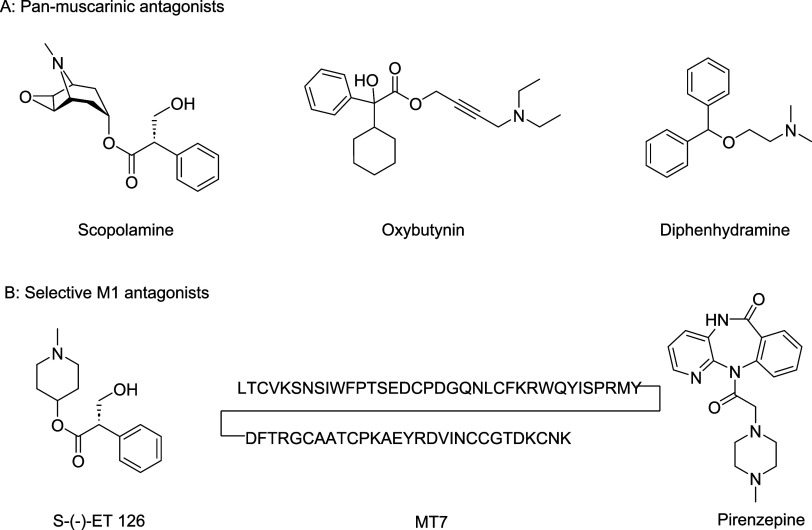
Pan-muscarinic antagonists (A) and selective M_1_ antagonists (B).

The ubiquity of mAChRs throughout biological compartments and their highly conserved orthosteric binding pocket pose a significant challenge in the development of highly selective therapeutic agents.^11^ Notably, most known muscarinic antagonists, such as scopolamine, oxybutynin, and diphenhydramine, are nonselective (Figure 1). Moreover, moderately selective M_1_ receptor antagonists, such as pirenzepine, have limited utility in treating CNS disorders due to poor blood-brain barrier penetration. However, pirenzepine shows promise in the treatment of neuropathic diseases of the PNS.^9,12^ Conversely, S-(−)-ET 126 (Figure 1), an analog of scopolamine, is a brain-penetrant compound with moderate selectivity for M_1_ over M_2_ and M_3_.^13^ However, its affinity for M_4_ and M_5_ remains unclear. MT7, a 65-amino-acid peptide with over 1000-fold selectivity for M_1_ over M_2–5_, was derived from the venom of the green mamba snake.^14^ The peptidic nature of MT7, unfortunately, makes it an unlikely drug candidate.

Our lab has been at the forefront of developing selective M_1_ antagonists for over a decade, starting with the Molecular Libraries Screening Center Network (MLPCN) development program, supported by the NIH Molecular Libraries Roadmap. This program led to the discovery of the highly selective M_1_ antagonist, **ML012** (**1**), which exhibited impressive selectivity for M_1_, ranging from 45- to 159-fold over M_2–5_ (Figure 2).^15^ In a rodent model, **ML012** ameliorated pilocarpine-induced seizures, a condition caused by the stimulation of M_1_ mAChR subtype receptor.^16^ Moreover, **ML012** mitigated pilocarpine-induced seizures without adversely affecting memory, highlighting the superior potential of selective M_1_ antagonists over pan-muscarinic antagonists, like scopolamine, for therapeutic development.^15^ Encouraged by these results, we initiated a hit-to-lead optimization campaign using an iterative parallel synthesis approach, resulting in the development of the isoquinoline sulfonamide **VU0415248** (**2**), an M_1_ antagonist with improved selectivity over M_2–5_.^16^ Further optimization led to the discovery of the more potent M_1_ antagonist **VU0452865** (**3**) without affecting M_1_ selectivity.^17^ Unfortunately, **VU0415248** and **VU0452865** exhibited high predicted rat hepatic clearance (CL_hep_) values of 68.6 mL/min/kg and 49.5 mL/min/kg, respectively. Subsequent efforts led by Consortium Pharmaceuticals (formerly PIPELINE Therapeutics) to explore this chemical space resulted in the development of PIPE-359 and later PIPE-307 (structure undisclosed), which was in Phase 2 clinical trials for patients with relapsing-remitting multiple sclerosis (RRMS).^18–20^ While this compound was tolerated and showed an acceptable safety profile at all doses tested, it did not demonstrate any significant change to visual function when binocular 2.5% low contrast letter acuity was assessed.^21^ Herein, we provide an update on our efforts to develop a viable *in vivo* tool as part of our ongoing pursuit in the discovery and development of M_1_ antagonists. Modifications were made to the western aryl, core, and eastern aza-heterocycle regions of the **VU0452865** scaffold, resulting in the development of potent and selective M_1_ antagonists (Figure 3).

**Figure 2:**
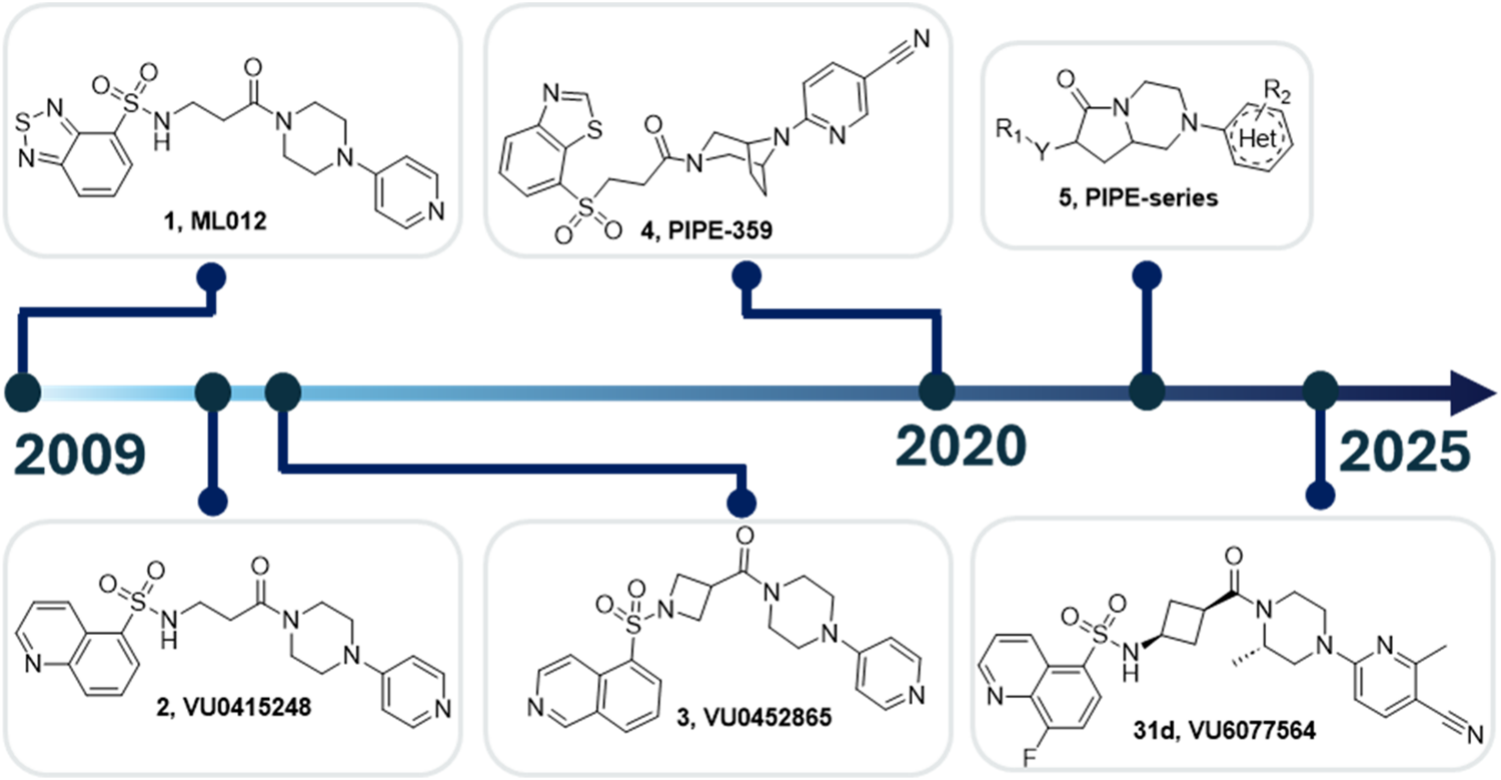
Timeline for the development of selective M_1_ antagonists.

**Figure 3:**
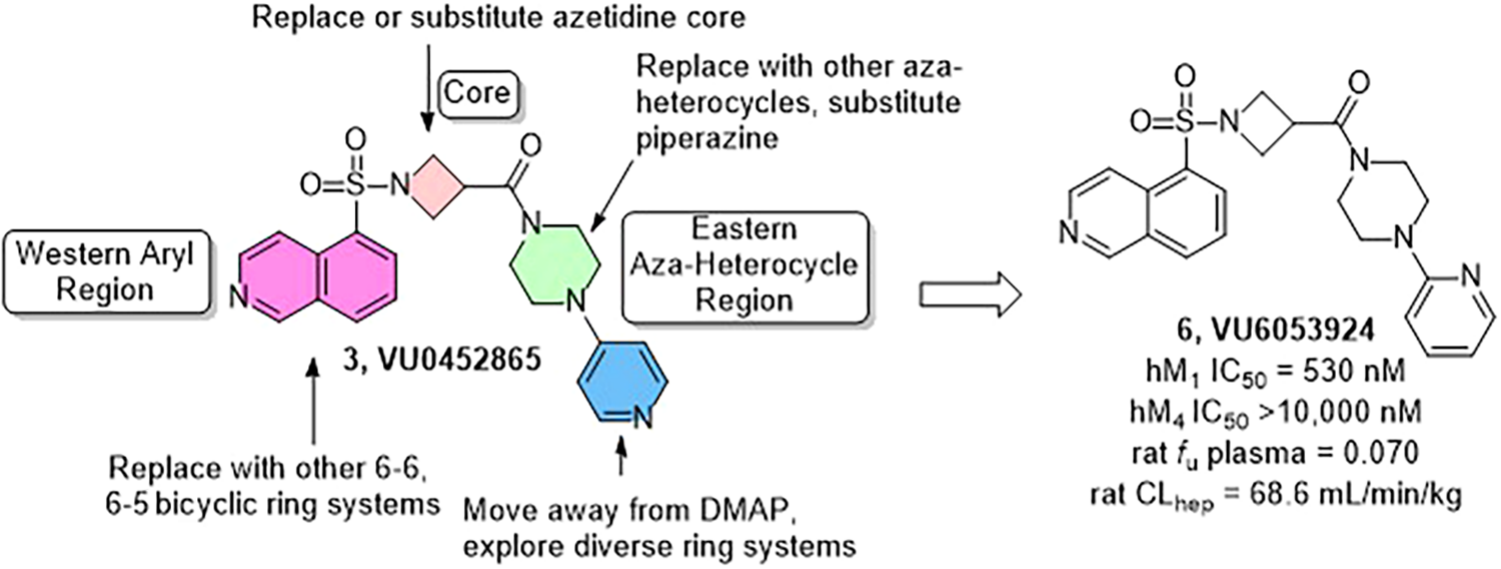
SAR studies strategy to improve **VU0452865**.

## Results and Discussion

Previously, our work toward the development of **VU0452865** (**3**) featured a 4-pyridyl group in the eastern region of our scaffolds. Concerned over the presence of a nucleophilic 4-dimethylaminopyridine (DMAP)-like moiety in our scaffold, we synthesized the 2-pyridyl analog **6** (Figure 3). While a 5-fold loss in potency was observed with this change (hM_1_ IC_50_ = 110 nM vs hM_1_ IC_50_ = 530 nM, respectively), both compounds display similar *in vitro* properties. Based on these results, we further explored structure–activity relationships (SAR) around **6**.

The synthesis of compounds **6**, **10**, and **11** began with the coupling of corresponding commercially available carboxylic acids (**7**) with the commercially available 2-(piperidin-4-yl) pyridine (**8**) (Scheme 1A). Following a Boc-deprotection with TFA in DCM, azetidines **9** could undergo sulfonamide formation with isoquinoline-5-sulfonyl chloride to afford analogs **6**, **10**, and **11**. For the synthesis of cyclobutyl analogs **15** and **16** (Scheme 1B), isoquinoline-5-sulfonyl chloride hydrochloride (**12**) was first treated with sodium sulfite and sodium bicarbonate to generate the corresponding sulfinate salt. The crude sulfinate salt was then reacted with cis or trans bromocyclobutanes **13** to give the corresponding sulfones. The ester moieties were then saponified to afford carboxylic acids **14**, which were then coupled with piperazine **8** under standard HATU conditions to furnish analogs **15** and **16**.

**Scheme 1:**
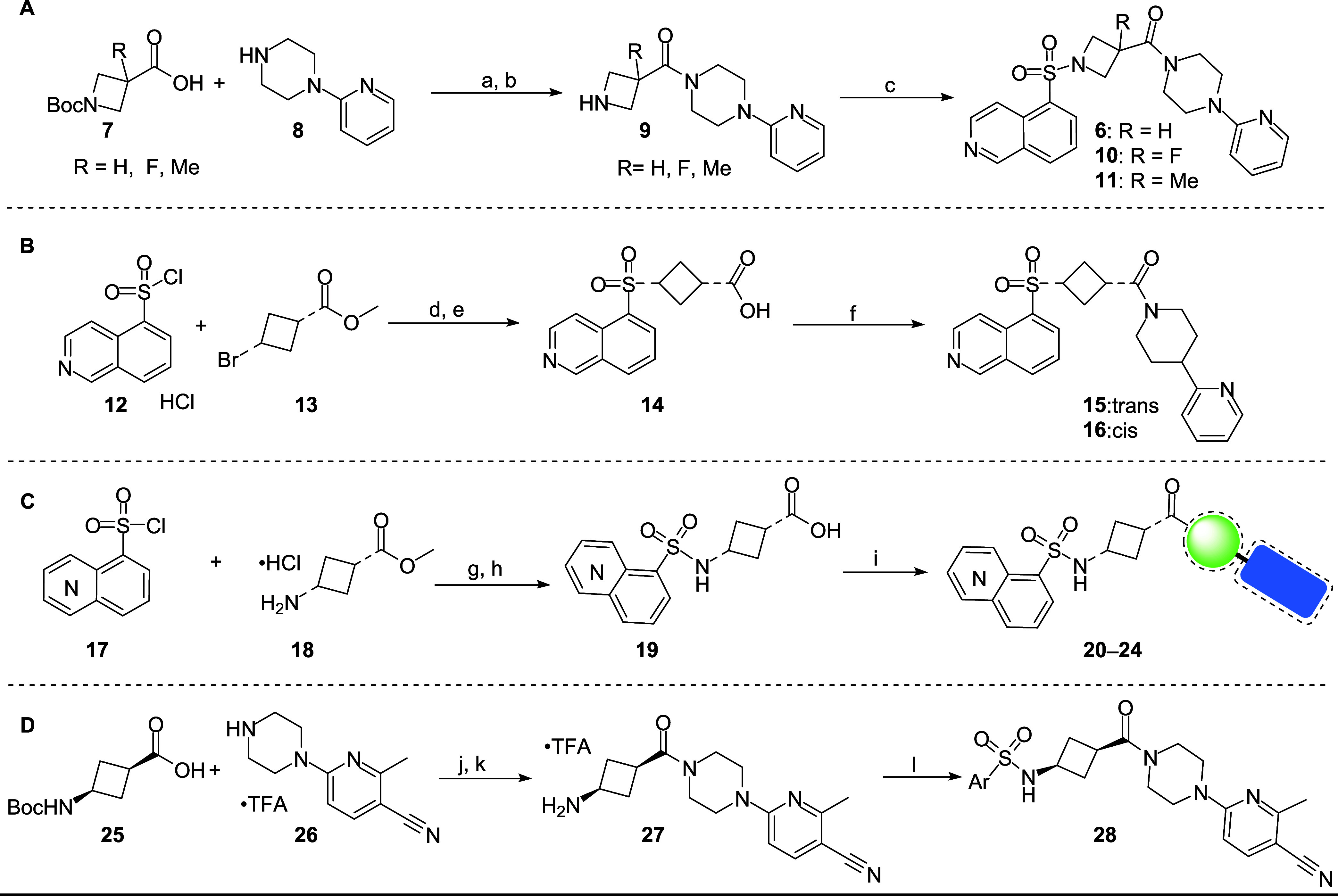
Synthesis of M_1_ Antagonist Analogs **6**, **10**, **11**, **15**, **16**, **20**–**24**, and **28** ^a^

a Reagents and conditions: (a) HATU, DIPEA, DMF, 17 h; (b) TFA, DCM, 2 h, quant. over two steps; (c) sulfonyl chloride, DIPEA, DCM, 2 h, 22**–**70%; (d) Na_2_SO_3_, NaHCO_3_ H_2_O, DMSO, microwave irradiated at 120 °C, 1 h, then DMF, alkyl-bromide, Cs_2_CO_3_, 17 h; (e) NaOH, DMF, 4 h, 26**–**32% over three steps; (f) HATU, DIPEA, DMF, 2 h, 26**–**53%; (g) DIPEA, DCM, 17 h, 99% (h) NaOH, 1,4-dioxane, 17 h, 99%; (i) aza-heterocycle, HATU, DIPEA, DMF, 2 h, 34**–**95%; (j) HATU, DIPEA, DMF, 17 h, 36%; (k) TFA, DCM, 4 h, quantitative; (l) aryl sulfonyl chloride, DIPEA, DCM, 1**–**4 h, 20**–**98%; for **28a**, perfluorophenyl quinoline-4-sulfonate, DIPEA, DCE, 50 °C, 1 h, 17%.

For the synthesis of cyclobutylsulfonamide (**20–24**, and **28**), we deployed iterative parallel synthesis approaches starting from commercially available amines **18** (Scheme 1C) or carboxylic acid **25** (Scheme 1D). Briefly, aryl sulfonyl chloride **17** was reacted with **18** followed by saponification to obtain the carboxylic acid **19**. The carboxylic acid was then coupled with various substituted aza-heterocycles to obtain the final compounds **20–24**. Alternatively, **25** was subjected to HATU amide coupling conditions with aza-heterocycle **26**, followed by subsequent Boc-deprotection with TFA to obtain the TFA salt **27**. Finally, intermediate **27** was subjected to sulfonation conditions with the corresponding aryl sulfonyl chlorides to give analogs **28**.

With final compounds in hand, analogs were screened against human M_1_ (hM_1_) in a calcium mobilization assay to determine their potency. Attempts to functionalize the azetidine ring were unsuccessful as both fluorination (**10**) and methylation (**11**) resulted in >3-fold reduction in potency (hM_1_ IC_50_ = 2,440 nM and 1,850 nM, respectively) (Table 1). Next, we explored the effect of introducing cis–trans geometric isomerism. Unfortunately, attempts to replace the azetidine moiety with cyclobutane (compounds **15** and **16**) or cyclobutylamine (compounds **20** and **21**) groups resulted in weak M_1_ antagonist activity (hM_1_ IC_50_ *s* > 10 μM).

**Table 1:**
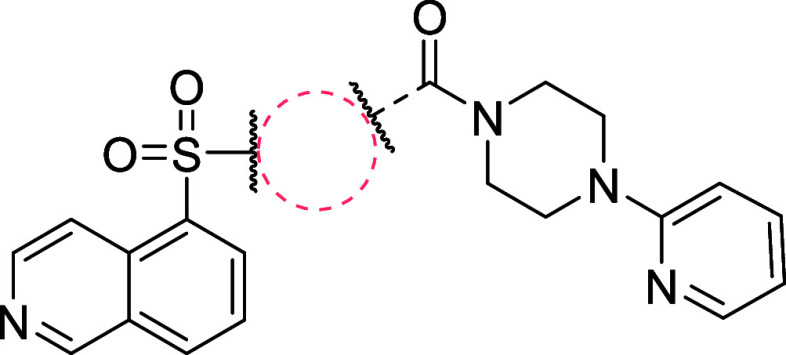

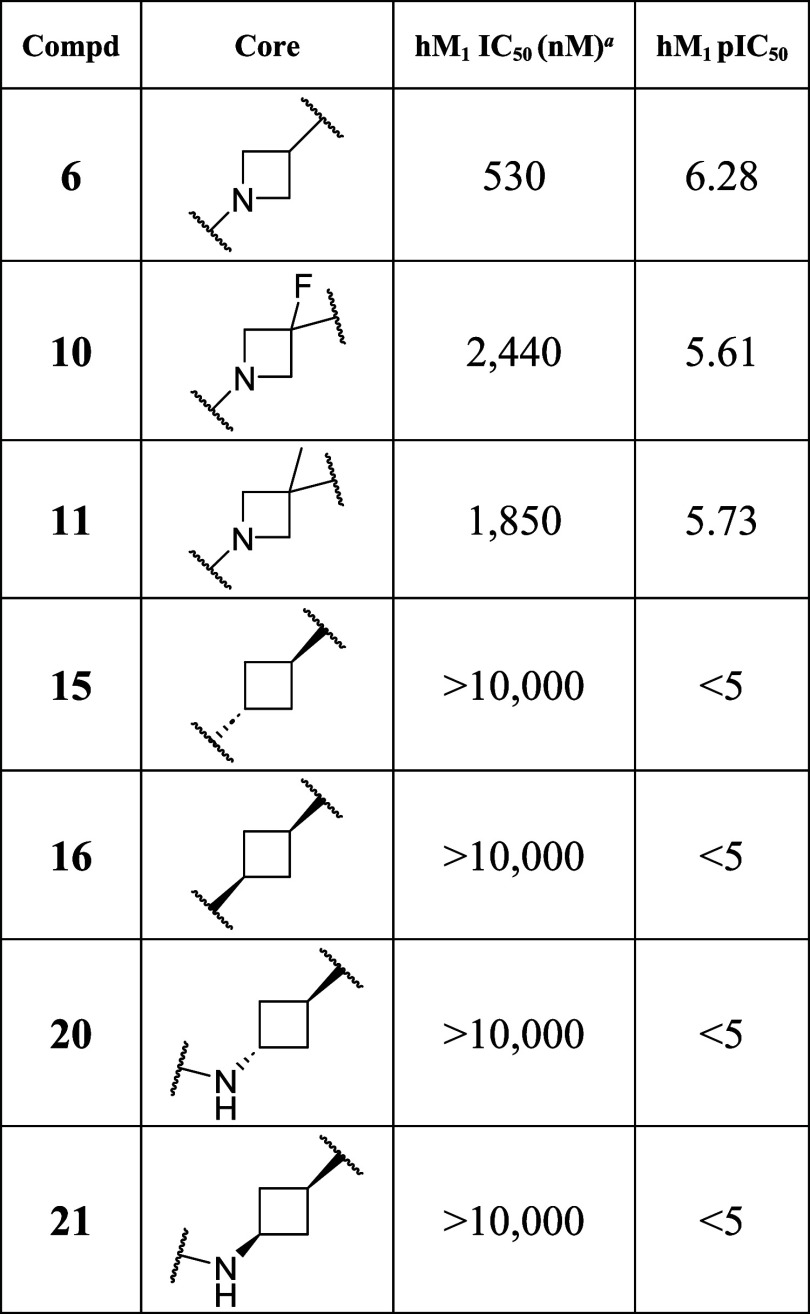
Structure and M_1_ Antagonist Activity of **6** and Its Analogs^a^

a Calcium mobilization assays with hM_1_-CHO cells performed in the presence of an EC_80_ fixed concentration of acetylcholine, *n* = 1–3 independent experiments in triplicate.

Assessment of historical SAR showed that **VU0415248** (**2**) contains a quinoline-5-sulfonamide rather than the isoquinoline-5-sulfonamide found in **VU0452865** (**3**). Based on these results, we synthesized quinoline-5-sulfonamide analogs of **22a** and **22b** (Figure 4). While compound **22a** displayed weak M_1_ antagonism (hM_1_ IC_50_ > 10 μM), compound **22b** displayed >3-fold improvement in potency for M_1_ (hM_1_ IC_50_ = 138 nM) in comparison to **VU0415248** (**2**) and **VU0452865** (**3**). Additionally, compound **22b** exhibited excellent selectivity for M_1_ over M_2–5_ (hM_2_ IC_50_ = 7.7 μM, hM_3–5_ IC_50_ > 10 μM). However, **22b** was found to have high predicted hepatic clearance in rat (rCL_hep_ = 63.0 mL/min/kg).

**Figure 4:**
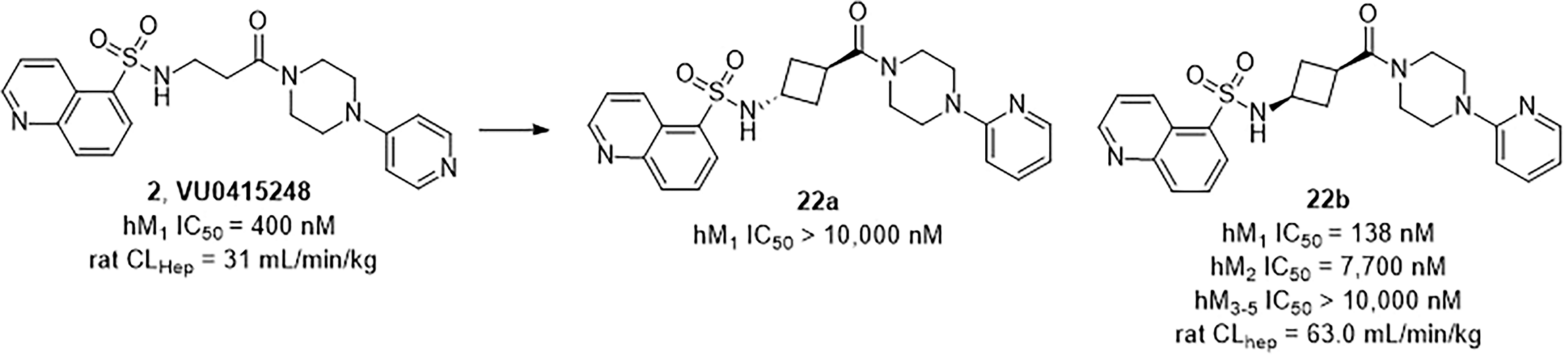
Impact of quinoline-5-sulfonamides on hM_1_ potency and *in vitro* clearance.

To better understand the major metabolic liabilities associated with **22b** to help direct SAR, a soft spot analysis was conducted in rat S9 fractions. While 59% of **22b** remained unchanged after incubation, a total of five metabolites were identified (Figure 5). Metabolite M467b, arising from the oxidation of the quinoline moiety was the most abundant (17%), followed by metabolite M515 (10%). Overall, the results indicated that oxidation of the 5-quinoline and 2-pyridylpiperazine moieties represented the primary source of metabolic liability, drawing our focus to these two regions of the molecule.

**Figure 5:**
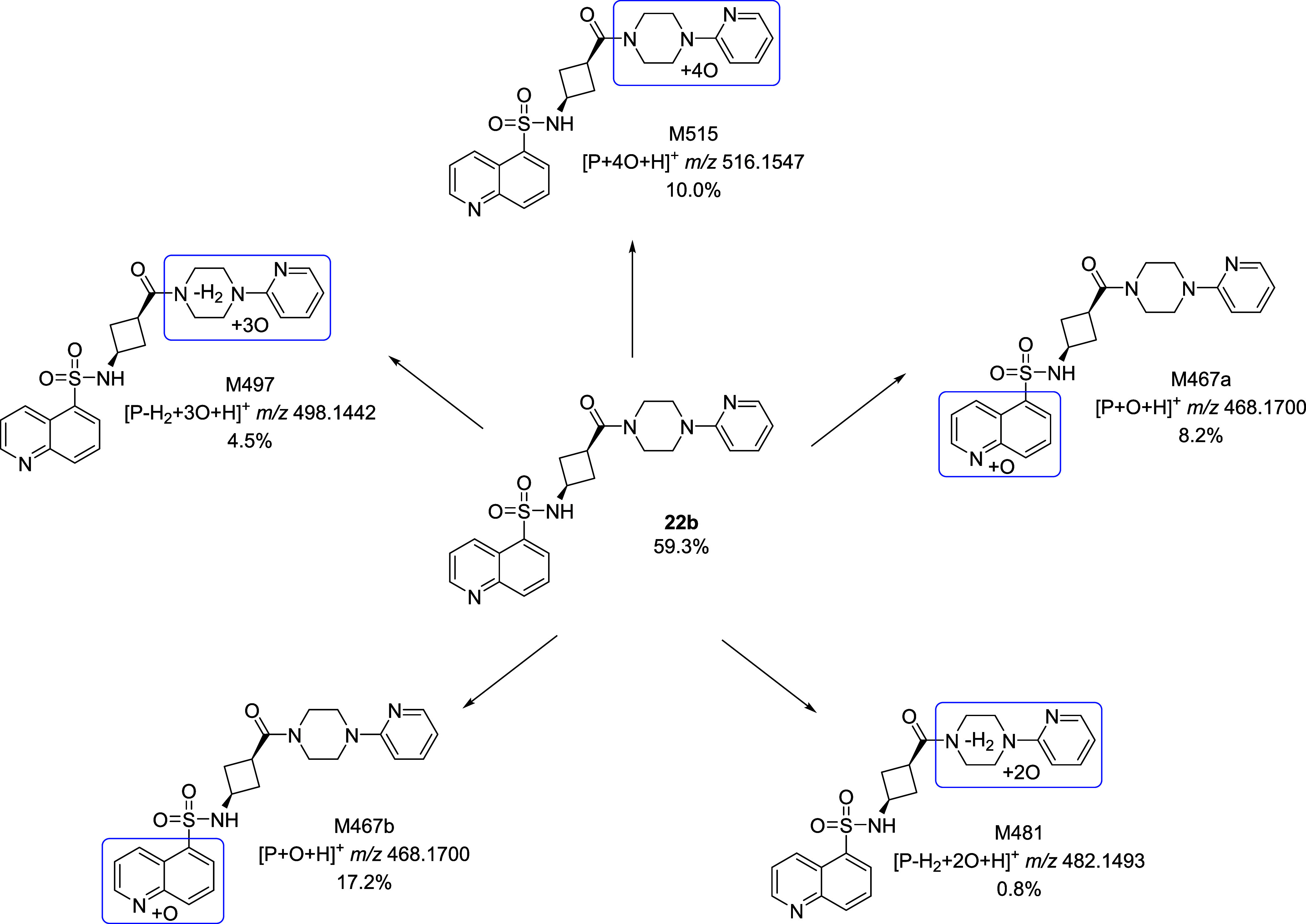
Soft spot analysis of **22b** in rat S9 fractions.

Based on the results of the soft spot analysis of **22b**, we first attempted to functionalize or replace the piperazine ring with other aza-heterocycles to block metabolism of this moiety (Table 2). Octa-deuteration of the piperazine ring (**23a**) resulted in a 6-fold loss in potency for M_1_ (hM_1_ IC_50_ = 820 nM). Methylation of the piperazine ring (analogs **23b–e**) was not tolerated, resulting in >8-fold loss in potency (hM_1_ IC_50_ *s* > 1.0 μM). While replacing the piperazine moiety with a piperidine (**23f**) also resulted in >20-fold loss in potency (hM_1_ IC_50_ = 3.2 μM), the piperazine isostere 3,7-diazabicyclo[3.3.0]­octane analog **23g** showed excellent potency for M_1_ (hM_1_ IC_50_ = 70 nM). However, like **22b**, the predicted rat hepatic clearance for **23g** was high (rCL_hep_ = 63.0 mL/min/kg). The 3,9-diazabicyclo[4.2.1]­nonane analog **23h** was equipotent (hM_1_ IC_50_ = 150 nM) to **22b** but also suffered from high predicted rat hepatic clearance (rCL_hep_ = 68.0 mL/min/kg). Additionally, **23h** was screened against other mAChR subtypes, and the results (hM_2_ IC_50_ = 890 nM, hM_4_ IC_50_ > 10 μM) indicated that while a bridged ring system can serve as a bioisostere for the piperazine ring, it reduces selectivity against M_2_ in this scaffold.

**Table 2:**
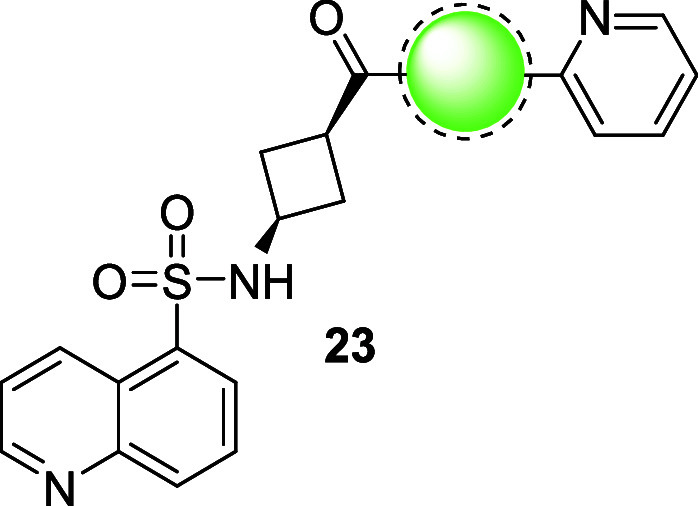

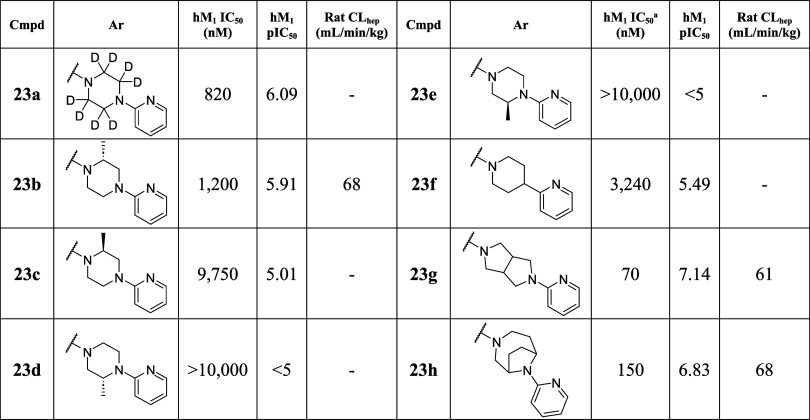
Structure and Activity of M_1_ Antagonist Analogs (**23a–h**) with Replacement and Substitution of the Piperazine Core^a^

a Calcium mobilization assays with hM_1_-CHO cells performed in the presence of an EC_80_ fixed concentration of acetylcholine, *n* = 1–3 independent experiments in triplicate.

As functionalization and replacement of the piperazine ring did not afford any analogs with improved microsomal stability, we next focused our attention on exploring SAR around the 2-pyridine ring (Table 3). While the 3-pyridine analog **24a** displayed acceptable potency for M_1_ (hM_1_ IC_50_ = 96 nM), its predicted hepatic clearance in rat was also high (rCL_hep_ = 57.0 mL/min/kg). Substitution on the 2-pyridine at either the 5- or 6-positions (**24b**-**24e**) was well tolerated, generating compounds with IC_50_ *s* ≤ 105 nM. However, rat microsomal clearance remained high for all compounds in question (rCL_hep_ *s* ≥ 56.0 mL/min/kg). In this series, **24c** stood out to us for its excellent potency for M_1_ (hM_1_ IC_50_ = 5.8 nM). Additional substitution on the **24c** pyridine ring system was explored as a method for improving metabolic stability. Methylation at either the 3- or 4- positions (**24f** and **24g**) resulted in >50-fold loss in potency (hM_1_ IC_50_ *s* = 300 nM and 721 nM, respectively) with no improvement to rat hepatic clearance (rCL_hep_ *s* = 64.0 mL/min/kg and 60 mL/min/kg, respectively). Gratifyingly, methylation at the 6-position (**24h**) not only retained excellent potency (hM_1_ IC_50_ = 11 nM) but also modestly improved predicted rat hepatic clearance (rCL_hep_ = 41.0 mL/min/kg). Replacement of the **24h** methyl group with a trifluoromethyl group (**24i**) maintained good potency (hM_1_ IC_50_ = 20 nM), while incorporation of an additional methyl group (**24j**) to **24h** led to a 30-fold loss in potency (hM_1_ IC_50_ = 331 nM). Both transformations led to compounds with high predicted hepatic clearances in rat (rCL_hep_ *s* ≥ 59.0 mL/min/kg). Bicyclic ring systems mimicking the electronics of **24h** were also explored. The 1,5-naphthyridine **24k** showed weak potency for M_1_ (hM_1_ IC_50_ > 10 μM), whereas the 1,8-naphthyridine **24l** retained excellent potency for M_1_ (hM_1_ IC_50_ = 10 nM). The predicted hepatic clearance in rat for **24l** remained high (rCL_Hep_ = 59.0 mL/min/kg).

**Table 3:**
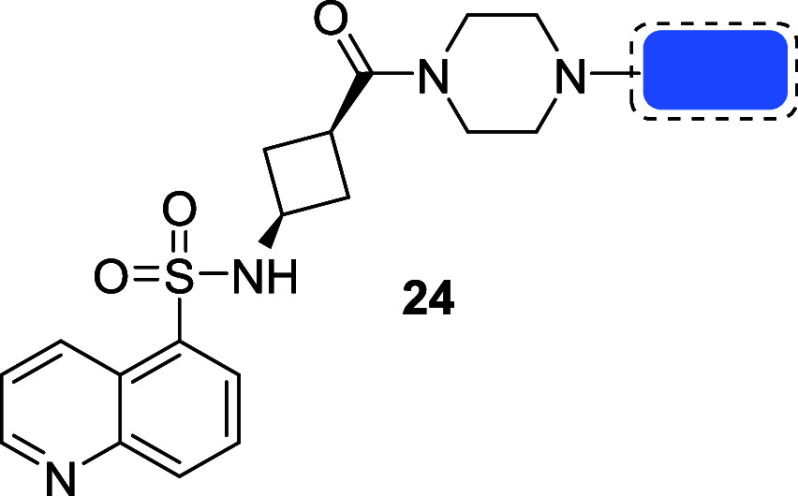

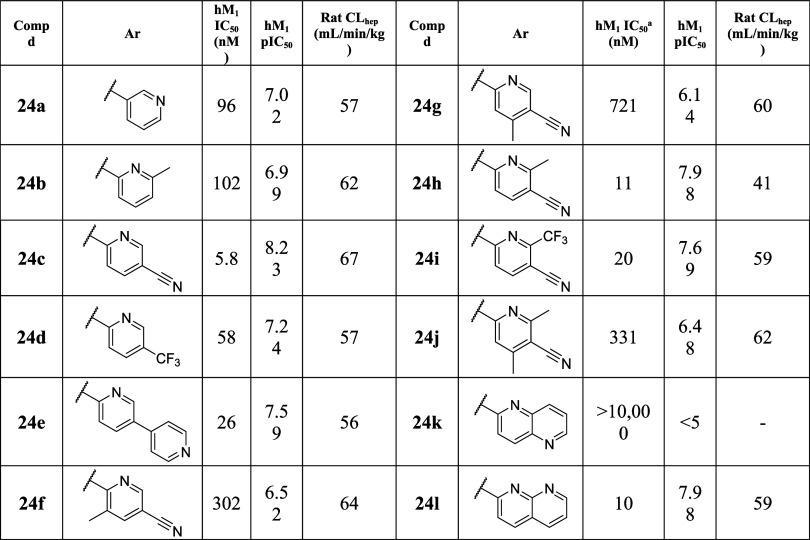
Structure and Activities of Selected M_1_ Antagonist Analogs (**24a–l**) with Changes to the Eastern Aryl Terminus^a^

a Calcium mobilization assays with hM_1_-CHO cells performed in the presence of an EC_80_ fixed concentration of acetylcholine, *n* = 1–3 independent experiments in triplicate.

Encouraged by its excellent potency and improved predicated hepatic clearance in rat, we explored optimization of the **24h** western aryl ring (Table 4). Replacement of the 5-quinoline ring with either a 4-quinoline (**28a**) or other bicyclic ring systems (**28b–d**) was well tolerated with hM_1_ IC_50_ *s* < 150 nM; however, high predicted hepatic clearance in rat persisted (rCL_hep_ *s* ≥ 51.0 mL/min/kg). Substitution at the 8-position **(28e)** of the 5-quinoline ring of **24h** with a methyl group led to >170-fold loss in potency (hM_1_ IC_50_ = 1.9 μM). On the other hand, fluorination at this same position (**28f**) proved more promising, generating a highly potent compound (hM_1_ IC_50_ = 71 nM) while displaying similar rat predicted hepatic clearance to **24h** (rCL_hep_ = 43.0 mL/min/kg).

**Table 4:**
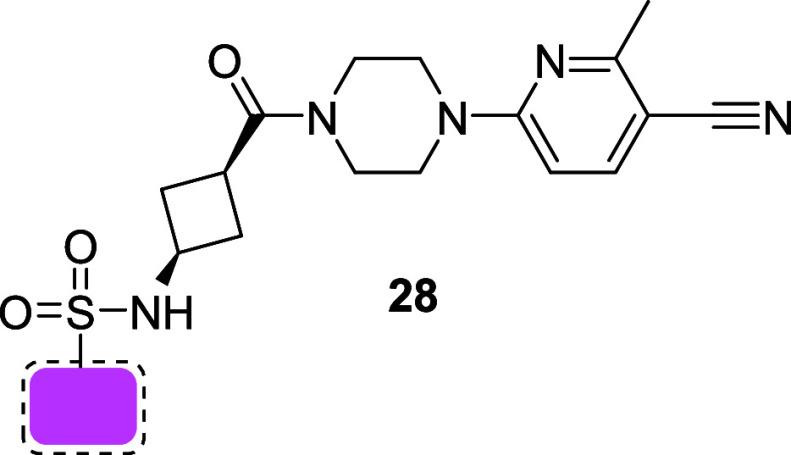

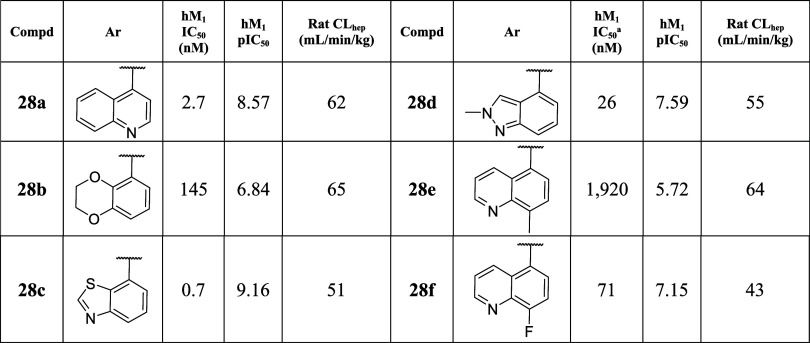
Structure and Activities of Selected M_1_ Antagonist Analogs (**28a–f**), Exploing the Western Aryl Terminus of Compound **24h** ^a^

a Calcium mobilization assays with hM_1_-CHO cells performed in the presence of an EC_80_ fixed concentration of acetylcholine, *n* = 1–3 independent experiments in triplicate.

Intrigued by the results with **28f**, we revisited a methyl scan of the piperazine core, (Table 5). The synthesis of compounds **31a–d** is outlined in Scheme 2, using the iterative parallel synthesis approaches described earlier. Methylation of the piperazine led to >5-fold loss in potency for **31a–c** (hM_1_ IC_50_s = 390 nM**–**1,130 nM). However, this loss in potency was offset by a modest improvement in *in vitro* rat clearance (rCL_hep_ *s* = 34.0**–**38.0 mL/min/kg). Compound **31d** was an outlier, showing ∼2-fold improvement in potency for M_1_ (hM_1_ IC_50_ = 31 nM) compared to **28f** while maintaining moderate predicted hepatic clearance in rat (rCL_hep_ = 40 mL/min/kg).

**Scheme 2:**
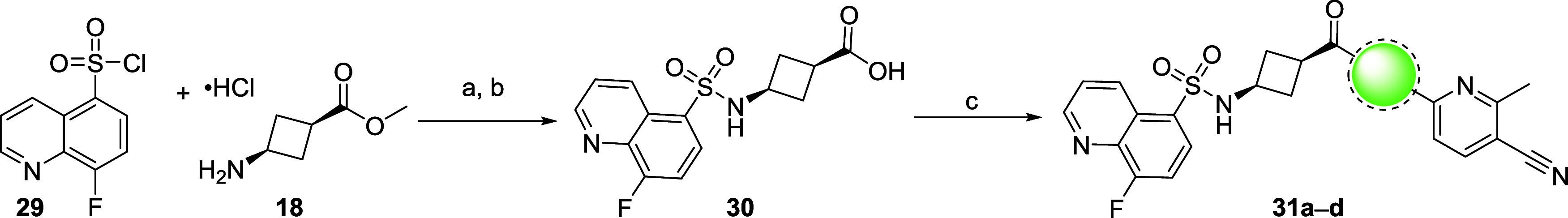
Synthesis of M_1_ Antagonist Analogs **31a**–**d** ^a^

a Reagents and conditions: (a) DIPEA, DCM, rt, 17 h, 64%; (b) 1:1 TFA/H_2_O, 100 °C, 1 h, 99%; (c) aryl piperazine, HATU, DIPEA, DMF, rt, 2 h, 35**–**47%.

**Table 5:**
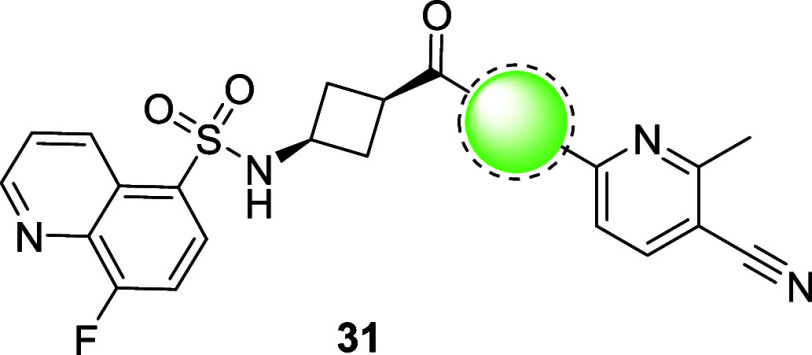

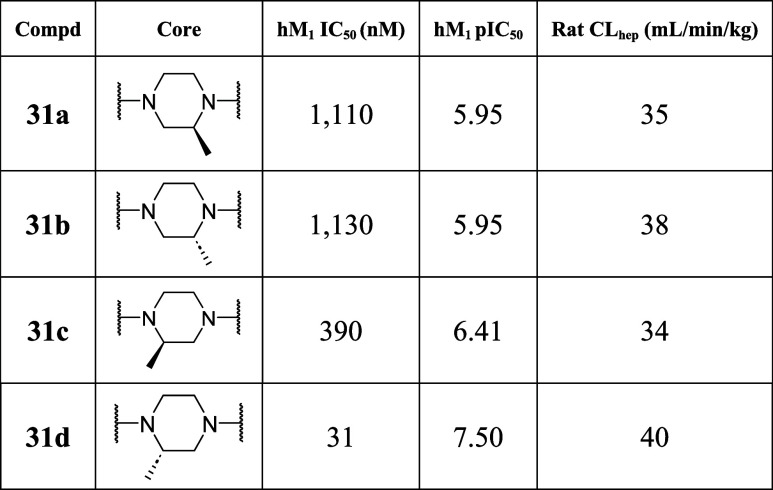
Structure and Activities of M_1_ Antagonist Analogs (**31a–d**) with Methyl Substitution of the Piperazine Core^a^

a Calcium mobilization assays in hM_1_-CHO cells were performed in the presence of an EC_80_ fixed concentration of acetylcholine, *n* = 2–3 independent experiments in duplicate or triplicate.

Compounds with hM_1_ IC_50_ *s* < 100 nM and predicted rat hepatic clearance values of ≤ 40 mL/min/kg were progressed for additional studies, narrowing our focus to compounds **24h**, **28f**, and **31d**. As **22b** showed weak activity toward hM_2_ (hM_2_ IC_50_ = 7.7 μM), we evaluated these three compounds against hM_2_ (Table 6). While compounds **28f** and **31d** showed excellent selectivity for hM_1_ versus hM_2_ (>140-fold selectivity), compound **24h** was only 12-fold selective for hM_1_ versus hM_2_. Based on these data, compounds **28f** and **31d** were advanced for further *in vitro* and *in vivo* studies (Table 7).

**Table 6:** Counter Screening of M_1_ Antagonists **24h**, **28f**, and **31d** against hM_2_ ^a^

	**VU6073552 (24h)**	**VU6074545 (28f)**	**VU6077564 (31d)**
hM_1_ IC_50_ (nM)	11	71	31
hM_2_ IC_50_ (nM)	132	>10,000	>10,000
hM_2_/hM_1_	12	∼141	∼323

a Calcium mobilization assays in hM_1_-CHO and hM_2/Gqi5_-CHO cells were performed in the presence of an EC_80_ fixed concentration of acetylcholine, *n* = 2–3 independent experiments in duplicate or triplicate.

**Table 7:** Additional *In Vitro* Pharmacology Characterization, *In Vitro* DMPK, and Rat *In Vivo* PK Profile of Analogs **28f** and **31d**

	**VU6074545 (28f)**	**VU6077564 (31d)**
MW	508.6	522.6
xLogP	1.78	2.28
tPSA (Å)	119.3	119.3
hM_1_ IC_50_ (nM)	71	31
rM_1_ IC_50_ (nM)	50.9	39.5
hM_1_ K_B_ (nM)	25	30
rM_1_ K_B_ (nM)	22	12
hM_2–5_ (μM)	>10	>10
**Rat** ***in vitro*** **PK parameters**		
rCL_int_ (mL/min/kg)	114	94
rCL_hep_ (mL/min/kg)	43.4	40.1
*f*_u,plasma_ (rat)^a^	0.020	0.019
*f*_u,brain_ (rat)^a^	0.036	0.031
MDCKII-MDR1 P-gp ER	48.0	46.6
***Rat in vivo*** **PK parameters**		
CL_p_ (mL/min/kg)^b^	17.6	71.7
*V*_ss_ (L/kg)	0.32	27.6
Elim. *t*_1/2_ (h)	0.33	35.2
*K*_p_ ^c^	0.03	0.08
*K*_p,uu_ ^d^	0.05	0.13

a *f*_*u*_ = Fraction unbound; equilibrium dialysis assay; brain = rat brain homogenates.

b Male Sprague–Dawley rats (*n* = 2); IV PK: 0.2 mg/kg, vehicle = 12% ethanol, 48% PEG400, 40% DMSO (0.5 mL/kg).

c *K*_p_ = total brain to total plasma ratio.

d *K*_p,uu_ = unbound brain (brain *f*_*u*_ × total brain) to unbound plasma (plasma *f*_*u*_ × total plasma) ratio.

Evaluation of **28f** and **31d** against the hM_3–5_ subtypes revealed excellent selectivity profiles, with IC_50_ *s* > 10 μM (Table 7). Compounds **28f** and **31d** were then tested in progressive fold-shift (PFS) experiments with Schild analysis to investigate their pharmacological activity at M_1_ in greater depth by determining their mechanism of action and affinity (*K*_B_) for the M_1_ receptor.^22^ These experiments demonstrated that **28f** and **31d** are reversible competitive antagonists (i.e., surmountable antagonism) and exhibit potent *K*_B_ values for human M_1_ (hK_B_ = 25 nM and 30 nM, respectively) (Figure S1). Additionally, **28f** and **31d** were also tested at rat M_1_ to assist in correlating data from rat models (rK_B_ = 22 nM and 12 nM, respectively) (Figure S2). Both compounds also showed modest free fraction in rat plasma (*f*_u,plasma_ ∼ 0.02) and rat brain homogenates (*f*_u,brain_ ∼ 0.031–0.036). When evaluated for rat *in vivo* pharmacokinetics (PK), **28f** displayed low clearance (CL_p_ = 17.6 mL/min/kg), a short elimination half-life (*t*_1/2_ ∼ 20 min), and low volume of distribution (*V*_ss_ = 0.32 L/kg). Compound **28f** also displayed low CNS penetration (rat brain:plasma *K*_p_ = 0.03) and low CNS distribution of unbound drug (*K*_p,uu_ = 0.05), which is in agreement with the high efflux ratio (ER = 48.0) obtained from *in vitro* studies utilizing MDCKII-MDR1 transfected cells.

Compound **31d** was found to have an *in vitro*-*in vivo* correlation (IVIVC) disconnect, displaying high *in vivo* clearance in rat (CL_p_ = 71.7 mL/min/kg) with a long elimination half-life of 35.2 h and high volume of distribution (27.6 L/kg). Compound **31d** also demonstrated low CNS penetration and distribution of unbound drug (*K*_p_ = 0.08 and *K*_p,uu_ = 0.13), which is aligned with the high efflux ratio observed *in vitro* (ER = 46.6). The relatively high topological polar surface area (tPSA) of 119.3 Å for **28f** and **31d** may contribute to the limited brain penetration observed with both compounds.

While **31d** displayed high *in vivo* clearance in rat, we were still encouraged by its excellent potency and selectivity for M_1_. We explored the neuroprotective potential of **31d** in neuropathic disease by studying its ability to elevate neurite outgrowth in cultured adult sensory neurons. We have previously shown that M_1_ antagonists can prevent or reverse key features of peripheral neuropathy in rodent models of diabetes, including loss of sensory nerve terminals, thermal hypoalgesia, and slowed nerve conduction.^5^ For this experiment various concentrations of **31d** were applied to adult rat DRG sensory neurons under defined conditions. This dissociated neuron culture system exhibits robust neurite outgrowth upon plating and the process of outgrowth and branching of axons is homologous to axonal sprouting that occurs in target tissues during innervation. Furthermore, this *in vitro* model permits the study of mechanisms of drug-induced axonal repair in response to disease states such as diabetes and chemotherapy treatment that trigger a distal dying-back of axons in the peripheral tissues.^17,23^ Sensory neurons were treated for 42 h with a range of **31d** concentrations in the presence of a low dose cocktail of neurotrophic factors (to provide optimal conditions for survival and growth and mimic the milieu *in vivo*) (Figure 6). A medium dose cocktail of neurotrophic factors was added in a separate group and acted as an internal control to judge the level of response between independent experiments. An approximate 2-fold maximal induction of neurite outgrowth was observed at 1000 nM of drug compared with low dose growth factor treatment. A statistically significant 1.7-fold elevation of total neurite outgrowth was induced by 100 nM and 300 nM of **31d**. The ED_50_ for elevation of neurite outgrowth by **31d** was determined as 55 nM (Figure S3), which aligns well with the rat functional potency and binding affinity (rat IC_50_ = 39.5 nM and rat *K*_B_ = 12 nM). There was no effect on the survival of sensory neurons across the full range of concentrations of **31d**.

**Figure 6:**
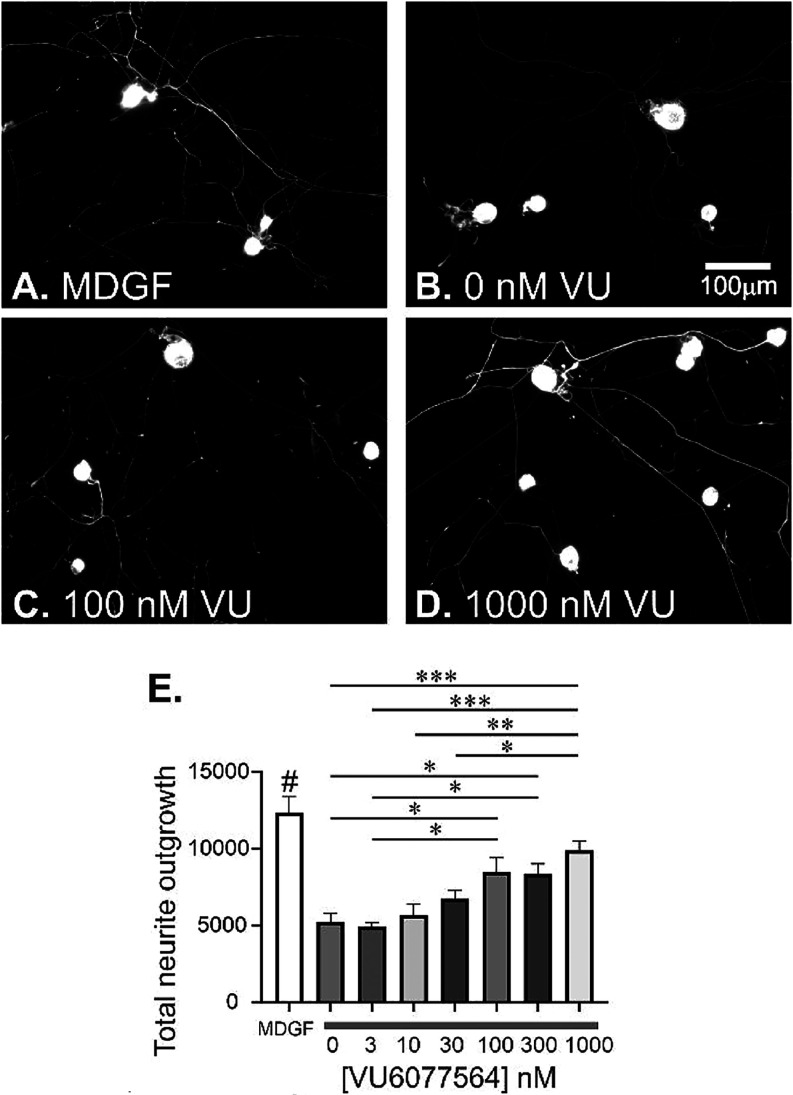
Effect of increasing doses of **VU6077564** (**31d**) on neurite outgrowth of adult rat sensory neurons. Dissociated DRG sensory neurons were cultured for 42 h under defined conditions in the presence of a low dose cocktail of growth factors (LDGF). Neurons were exposed to a range of concentrations of **VU6077564** (VU) and a separate group were treated with a medium dose cocktail of growth factors (MDGF) as an internal control. Neurons were fixed, stained for neuron-specific peripherin and total neurite outgrowth assessed. Fluorescent images from cultures are shown in (A) MDGF, (B) 0 nM drug (LDGF alone), (C) 100 nM **VU6077564** and (D) 1000 nM **VU6077564**. Bar = 100 μm. In (E) is a bar chart showing data for total neurite outgrowth. Values are means ± SEM, *n* = 6 replicate cultures. Data analyzed by one-way ANOVA with Tukey’s *post hoc* test.* *P* < 0.05, ** *P* < 0.01, *** *P* < 0.001, # significantly different at *P* < 0.01 vs all groups except 1000 nM drug.

## Conclusion

Overall, we identify **VU6077564** (**31d**) as a lead compound within a new series of potent and highly selective cyclobutylsulfonamide-based M_1_ antagonists. **VU6077564** displays high *in vivo* clearance in rat and is peripherally restricted (*K*_p_ = 0.08, *K*_p,uu_ = 0.13, ER = 46.6). Herein, we demonstrate **VU6077564** promotes neurite outgrowth in cultured adult DRG sensory neurons, further supporting the therapeutic potential of peripherally restricted and selective M_1_ antagonists for therapy in axonal dying-back diseases of the PNS, such as diabetic neuropathy, chemotherapy-induced peripheral neuropathy, and HIV-neuropathy. Moreover, valuable lessons have been gleaned from this work that inform strategies to improve *in vivo* clearance, the details of which will be reported in due course.

## Methods

### General Information^24^

All chemicals were purchased from commercial vendors and used without further purification. All NMR spectra were recorded on a 400 MHz AMX Bruker NMR spectrometer. ^1^H and ^13^C chemical shifts are reported in δ values in ppm downfield with the deuterated solvent as the internal standard. Low resolution mass spectra were obtained on an Agilent 6120/6150 or Waters QDa (Performance) SQ MS with ESI source. High resolution mass spectra were obtained on an Agilent 6540 UHD Q-TOF with ESI source. Normal phase column chromatography was performed on a Teledyne ISCO CombiFlash Rf+ system. For compounds that were purified on a Gilson preparative reversed-phase HPLC, the system comprised of a 333 aqueous pump with solvent selection valve, 334 organic pump, GX 271 or GX-281 liquid hander, two column switching valves, and a 155 UV detector. Solvents for extraction, washing and chromatography were HPLC grade. All final compounds were found to be >95% pure by HPLC-MS analysis.

#### Synthesis. 2-Methyl-6-(piperazin-1-yl)­nicotinonitrile; 2,2,2-trifluoroacetate (**26**)

Step 1: To a solution of 6-chloro-2-methylnicotinonitrile (60 mg, 0.39 mmol) in MeCN (2.6 mL) was added *tert*-butyl piperazine-1-carboxylate (110 mg, 0.59 mmol) and DIPEA (0.137 mL, 0.79 mmol). After stirring at 80 °C for 17 h, the reaction mixture was cooled to rt, diluted with water, and extracted with DCM (3×). The combined organics were passed through a phase separator and concentrated. Purification via normal-phase column chromatography on silica gel (0–50% EtOAc/hexanes) afforded *tert*-butyl 4-(5-cyano-6-methylpyridin-2-yl)­piperaine-1-carboxylate as a white solid (110 mg, 93% yield). ^1^H NMR (400 MHz, CD_3_OD) δ 7.66 (d, *J* = 9.0 Hz, 1H), 6.68 (d, *J* = 9.0 Hz, 1H), 3.75–3.67 (m, 4H), 3.55–3.48 (m, 4H), 2.52 (s, 3H), 1.48 (s, 9H). HRMS (Q-TOF, ES+): Calculated for C_16_H_22_N_4_O_2_ (M + H)^+^, 303.1816**;** Observed, 303.1822. Step 2: To a solution of *tert*-butyl 4-(5-cyano-6-methylpyridin-2-yl)­piperaine-1-carboxylate (100 mg, 0.33 mmol) in DCM (2 mL) was added TFA (0.215 mL, 2.81 mmol). After stirring for 17 h, the mixture was concentrated *in vacuo* and carried forward without further purification in quantitative yield. ^1^H NMR (400 MHz, D_2_O) δ 7.89 (d, *J* = 9.1 Hz, 1H), 6.89 (d, *J* = 9.1 Hz, 1H), 3.99 (dd, *J* = 5.4, 5.3 Hz, 4H), 3.39 (dd, *J* = 5.4, 5.3 Hz, 4H), 2.61 (s, 3H). HRMS (Q-TOF, ES+): Calculated for C_11_H_14_N_4_ (M + H)^+^, 203.1291**;** Observed, 203.1296.

#### 6-(4-((1*s*,3*s*)-3-Aminocyclobutane-1-carbonyl)­piperazin-1-yl)-2-methylnicotinonitrile 2,2,2-trifluoroacetate (**27** )

Step 1: To a solution of **26** (107 mg, 0.33 mmol) in DMF (1.4 mL) was added *cis*-3-(*tert*-butyoxycarbonylamino)­cyclobutanecarboxylic acid (60 mg, 0.28 mmol), HATU (159 mg, 0.42 mmol), and DIPEA (0.146 mL, 0.84 mmol). After stirring a rt for 17 h, the reaction mixture was syringe filtered and purified using RP-HPLC (20–60% ACN/0.5% aqueous NH_4_OH) to give *tert*-butyl ((1*s*,3*s*)-3-(4-(5-cyano-6-methylpyridin-2-yl)­piperazine-1-carbonyl)­cyclobutyl)­carbamate as a white solid (40 mg, 36% yield). ^1^H NMR (400 MHz, CD_3_OD) δ 7.68 (d, *J* = 8.9 Hz, 1H), 6.69 (d, *J* = 9.0 Hz, 1H), 4.06–3.94 (m, 1H), 3.77–3.63 (m, 6H), 3.61–3.55 (m, 2H), 3.12–3.04 (m, 1H), 2.56–2.47 (m, 5H), 2.17–2.06 (m, 2H), 1.43 (s, 9H). HRMS: (Q-TOF, ES+): Calculated for C_21_H_29_N_5_O_3_ (M+H)^+^, 400.2343**;** Observed, 400.2351. Step 2: To a solution of *tert*-butyl ((1*s*,3*s*)-3-(4-(5-cyano-6-methylpyridin-2-yl)­piperazine-1-carbonyl)­cyclobutyl)­carbamate (37 mg, 0.092 mmol) in DCM (0.5 mL) was added TFA (0.06 mL, 0.7835 mmol). After stirring at rt for 3 h, the mixture was concentrated *in vacuo* and carried forward without further purification in quantitative yield. ^1^H NMR (400 MHz, D_2_O) δ 8.02 (d, *J* = 9.5 Hz, 1H), 7.13 (d, *J* = 9.6 Hz, 1H), 3.93–3.73 (m, 9H), 3.45–3.31 (m, 1H), 2.73 (s, 3H), 2.70–2.61 (m, 2H), 2.44–2.32 (m, 2H). HRMS: (Q-TOF, ES+): Calculated for C_16_H_21_N_5_O (M + H)^+^, 300.1819**;** Observed, 300.1827.

#### *N*-((1*s*,3*s*)-3-(4-(5-Cyano-6-methylpyridin-2-yl)­piperazine-1-carbonyl)­cyclobutyl)-8-fluoroquinoline-5-sulfonamide (**28f**)

To a solution of **27** (38 mg, 0.092 mmol) in DCM (0.5 mL) was added 8-fluoroquinoline-5-sulfonyl chloride (25 mg, 0.10 mmol) and TEA (0.04 mL, 0.58 mmol). After stirring at rt for 1.5 h, the reaction mixture was diluted with water and extracted with DCM (3×). The combined organics were passed through a phase separator and concentrated. Purification via RP-HPLC (20–60% ACN/0.5% aqueous NH_4_OH) afforded an off-white solid (28 mg, 60% yield). ^1^H NMR (400 MHz, (CD_3_)_2_CO) δ 9.15 (ddd, *J* = 8.8, 1.6 Hz, *J*_*HF*_ = 1.7 Hz, 1H), 9.07 (dd, *J* = 4.1, 1.6 Hz, 1H), 8.32 (dd, *J* = 8.3 Hz, *J*_*HF*_ = 5.0 Hz, 1H), 7.79 (dd, *J* = 8.8, 4.1 Hz, 1H), 7.68 (d, *J* = 8.9 Hz, 1H), 7.67 (dd, *J* = 8.3 Hz, *J*_*HF*_ = 9.9 Hz, 1H), 7.32 (d, *J* = 9.1 Hz, 1H), 6.69 (d, *J* = 9.0 Hz, 1H), 3.86–3.73 (m, 1H), 3.69–3.59 (m, 4H), 3.58–3.53 (m, 2H), 3.49–3.42 (m, 2H), 2.96 (tt, *J* = 9.8, 7.9 Hz, 2H), 2.48 (s, 3H), 2.26–2.13 (m, 1H), 2.03–1.93 (m, 2H). ^13^C NMR (101 MHz, (CD_3_)_2_CO) δ 172.11, 161.92, 161.87 (d, *J*_*CF*_ = 263.3 Hz), 159.84, 152.32 (d, *J*_*CF*_ = 2.1 Hz), 141.74, 139.73 (d, *J*_*CF*_ = 11.7 Hz), 134.26 (d, *J*_*CF*_ = 2.5 Hz), 134.09 (d, *J*_*CF*_ = 4.9 Hz), 131.04 (d, *J*_*CF*_ = 9.5 Hz), 126.79 (d, *J*_*CF*_ = 2.5 Hz), 124.58, 119.19, 113.08 (d, *J*_*CF*_ = 20.1 Hz), 104.73, 96.39, 45.22, 45.13, 44.87, 41.97, 34.79, 34.76, 23.84. HRMS (Q-TOF, ES+): Calculated for C_25_H_25_FN_6_O_3_S (M + H)^+^, 509.1766**;** Observed, 509.1772.

#### *cis*-3-[(8-Fluoroquinolin-5-yl)­sulfonylamino]­cyclobutane-1-carboxylic acid (**30**)

Step 1: To a solution of *cis*-methyl 3-aminocyclobutanecarboxylate hydrochloride (371 mg, 2.24 mmol) and DIPEA (1.8 mL, 10.18 mmol) in DCM (10 mL) in an ice bath was added 8-fluoroquinoline-5-sulfonyl chloride (500 mg, 2.04 mmol). The resulting reaction mixture was allowed to warm to rt and stirred for 17 h. The reaction was then quenched with saturated aqueous NaHCO_3_ solution and extracted with DCM (2×). The combined organic layers were concentrated and purified by using normal-phase column chromatography on silica gel (10–100% EtOAc/hexanes) to provide *cis*-methyl 3-[(8-fluoroquinolin-5-yl)­sulfonylamino]­cyclobutane-1-carboxylate (440 mg, 64% yield) as a white solid. ^1^H NMR (400 MHz, CD_3_OD) δ 9.18 (ddd, *J* = 8.8, 1.6 Hz, *J*_*HF*_ = 1.6 Hz, 1H), 9.03 (dd, *J* = 4.3, 1.6 Hz, 1H), 8.31 (dd, *J* = 8.3 Hz, *J*_*HF*_ = 5.0 Hz, 1H), 7.82 (dd, *J* = 8.9, 4.3 Hz, 1H), 7.63 (dd, *J* = 8.3 Hz, *J*_*HF*_ = 9.9 Hz, 1H), 3.76–3.61 (m, 1H), 3.57 (s, 3H), 2.67 (tt, *J* = 10.0, 7.9 Hz, 1H), 2.22–2.14 (m, 2H), 1.96–1.86 (m, 2H). HRMS: (Q-TOF, ES+): Calculated for C_15_H_15_FN_2_O_4_S (M+H)^+^, 339.0809**;** Observed, 339.0814. Step 2: To *cis*-methyl 3-[(8-fluoroquinolin-5-yl)­sulfonylamino]­cyclobutane-1-carboxylate (170 mg, 0.5 mmol) was added water (1 mL) and TFA (1 mL, 13.06 mmol), and the mixture was stirred at 100 °C for 2 h. The solvent was removed *in vacuo* to afford a white powder that was carried forward without further purification 162 mg^1^H NMR (400 MHz, CD_3_OD) δ 9.18 (ddd, *J* = 8.8, 1.6 Hz, *J*_*HF*_ = 1.6 Hz, 1H), 9.03 (dd, *J* = 4.2, 1.5 Hz, 1H), 8.31 (dd, *J* = 8.3 Hz, *J*_*HF*_ = 4.9 Hz, 1H), 7.82 (dd, *J* = 8.9, 4.2 Hz, 1H), 7.63 (dd, *J* = 8.3 Hz, *J*_*HF*_ = 9.9 Hz, 1H), 3.68 (tt, *J* = 9.2, 7.5 Hz, 1H), 2.62 (tt, *J* = 10.0, 7.9 Hz, 1H), 2.32–2.06 (m, 2H), 1.97–1.84 (m, 2H). HRMS: (Q-TOF, ES+): Calculated for C_14_H_13_FN_2_O_4_S, (M + H)^+^, 325.0653**;** Observed, 325.0656.

#### (*S*)-2-Methyl-6-(3-methylpiperazin-1-yl)­nicotinonitrile; (2,2,2-trifluoroacetate)

*Tert*-butyl (2*S*)-2-methyl-1-piperazinecarboxylate (74 mg, 0.37 mmol) was dissolved in MeCN (1 mL). Cesium carbonate (71 mg, 0.73 mmol) and 6-fluoro-2-methylnicotinonitrile (50 mg, 0.37 mmol) were added sequentially, and the reaction mixture was refluxed for 17 h. The reaction mixture was diluted with water followed by extraction with DCM (2×). The combined organic layers were passed through a phase separator and concentrated. To the concentrated residue was added DCM (0.5 mL) and TFA (0.113 mL, 1.47 mmol). After stirring at rt for 2 h, solvents were removed *in vacuo*, and the sample carried forward without further purification as a brown solid (120.9 mg). ^1^H NMR (400 MHz, CD_3_OD) δ 7.76 (d, *J* = 8.9 Hz, 1H), 6.82 (d, *J* = 8.9 Hz, 1H), 4.66–4.55 (m, 2H), 3.48 (dt, *J* = 12.6, 2.8 Hz, 1H), 3.44–3.35 (m, 1H), 3.34–3.25 (m, 1H), 3.18 (td, *J* = 12.2, 3.3 Hz, 1H), 3.07 (dd, *J* = 14.4, 10.6 Hz, 1H), 2.55 (s, 3H), 1.39 (d, *J* = 6.6 Hz, 3H). HRMS (Q-TOF, ES+): Calculated for C_12_H_16_N_4_ (M + H)^+^, 217.1448**;** Observed, 217.1448.

#### *N*-((1*R*,3*s*)-3-((*S*)-4-(5-Cyano-6-methylpyridin-2-yl)-2-methylpiperazine-1-carbonyl)­cyclobutyl)-8-fluoroquinoline-5-sulfonamide (**31d**)

To a mixture of intermediate **30** (20 mg, 0.062 mmol), (*S*)-2-methyl-6-(3-methylpiperazin-1-yl)­nicotinonitrile; (2,2,2-trifluoroacetate) (20 mg, 0.062 mmol), and HATU (28 mg, 0.074 mmol) in DMF (1 mL) was added DIPEA (0.035 mL, 0.25 mmol). After stirring at rt for 2 h, the mixture was syringe filtered and purified using RP-HPLC (15–70% ACN/0.5% aqueous NH_4_OH) to give a white solid (15 mg, 47% yield). ^1^H NMR (400 MHz, CDCl_3_) (mixture of rotamers) δ 9.13–9.02 (m, 2H), 8.28 (dd, *J* = 8.3 Hz, *J*_*HF*_ = 4.9 Hz, 1H), 7.63 (dd, *J* = 8.7, 4.2 Hz, 1H), 7.58 (d, *J* = 8.9 Hz, 1H), 7.46 (dd, *J* = 8.3 Hz, *J*_*HF*_ = 9.4 Hz, 1H), 6.40 (d, *J* = 8.9 Hz, 1H), 6.20 (d, *J* = 9.6 Hz, 0.5H), 6.11 (d, *J* = 9.4 Hz, 0.5H), 4.84–4.72 (m, 0.5H), 4.48–4.34 (m, 1H), 4.24 (d, *J* = 13.4 Hz, 0.5H), 4.14 (d, *J* = 12.1 Hz, 0.5H), 4.06–3.90 (m, 1H), 3.79 (h, *J* = 8.2 Hz, 1H), 3.55–3.31 (m, 1H), 3.31–3.22 (m, 1H), 3.16–2.93 (m, 1.5H), 2.86 (dt, *J* = 17.7, 8.4 Hz, 1H), 2.57 (s, 3H), 2.39–2.26 (m, 2H), 2.19–2.00 (m, 2H), 1.13 (dd, *J* = 20.2, 6.7 Hz, 3H). ^13^C NMR (101 MHz, CDCl_3_) (mixture of rotamers) δ 172.2 (d, *J*_*CF*_ = 39.6 Hz), 161.8, 161.4 (d, *J*_*CF*_ = 265.5 Hz), 158.8, 151.4, 141.2, 138.9 (d, *J*_*CF*_ = 12.0 Hz), 133.5, 132.1 (d, *J*_*CF*_ = 3.3 Hz), 130.3 (d, *J*_*CF*_ = 9.5 Hz), 126.1 (d, *J*_*CF*_ = 2.8 Hz), 123.8, 118.7, 112.2 (d, *J*_*CF*_ = 20.1 Hz), 103.2 (d, *J*_*CF*_ = 7.1 Hz), 96.3, 49.5, 48.3, 48.0, 46.1, 44.9, 44.7, 44.4, 40.4, 36.8, 34.8, 34.5, 34.4, 34.2, 30.7, 30.2, 23.7, 17.6, 16.0. HRMS (Q-TOF, ES+): Calculated for C_26_H_27_FN_5_O_3_S (M + Na)^+^, 545.1742**;** Observed, 545.1741

### Molecular Pharmacology

#### Calcium Mobilization Assays

Compound-evoked decrease to an EC_80_ concentration of acetylcholine (ACh) in intracellular calcium were measured using Chinese hamster ovary (CHO) cells stably expressing human or rat muscarinic receptors (M_1_–M_5_; M_2_ and M_4_ cells were coexpressed with chimeric G_qi5_). The stable cells were cultured in F12 medium containing 10% fetal bovine serum, 20 mM HEPES, 100 units/mL antibiotics/antimycotic, 0.5 mg/mL G418, and 0.2 mg/mL hygromycin (M_2_ and M_4_ G_qi5_ coexpressing cells only). All reagents used were from Life Technologies (Carlsbad, CA) unless otherwise noted. Briefly, the day before the assay, cells (15,000 cells/20 μL/well) were plated in black-walled, clear-bottomed, 384 well plates (Greiner Bio-One, Monroe, NC) in the culture medium without G418 and hygromycin, and then incubated overnight at 37 °C in the presence of 5% CO_2_. The next day, calcium assay buffer (Hank’s balanced salt solution (HBSS), 20 mM HEPES, 2.5 mM Probenecid, 4.16 mM sodium bicarbonate (Sigma-Aldrich, St. Louis, MO)) was prepared to dilute compounds, agonists, and Fluo-4-acetomethoxyester (Fluo-4-AM), fluorescent calcium indicator dye. Compounds were serially diluted 1:3 or 1:5 into 10-point concentration response curves in DMSO using the Bravo Liquid Handler (Agilent, Santa Clara, CA), transferred to a 384 well daughter plates using an Echo acoustic liquid handler (Beckman Coulter, Indianapolis, Indiana), and diluted in assay buffer to a 2× final concentration. The agonist plates were prepared using acetylcholine (ACh, Sigma-Aldrich, St. Louis, MO) concentrations for the EC_20_, EC_80_, and EC_Max_ responses by diluting in assay buffer to a 5× final concentration. The 2× dye solution (2.3 μM) was prepared by mixing a 2.3 mM Fluo-4-AM stock in DMSO with 10% (w/v) pluronic acid F-127 in a 1:1 ratio in assay buffer. Using a microplate washer (BioTek, Winooski, VT), cells were washed with assay buffer 3 times to remove medium. After the final wash, 20 μL of assay buffer remained in the cell plates. Immediately, 20 μL of the 2× dye solution (final 1.15 μM) was added to each well of the cell plate using a Multidrop Combi dispenser (Thermo Fisher, Waltham, MA). After cells were incubated with the dye solutions for 45 min at 37 °C in the presence of 5% CO_2_, the dye solutions were removed and replaced with assay buffer using a microplate washer, leaving 20 μL of assay buffer in the cell plate, and the cell plate allowed to incubate for 10 min at 37 °C. The compound, agonist, and cell plates were placed inside the Functional Drug Screening System 7000 (FDSS7000, Hamamatsu, Japan) to measure the calcium flux. After establishment of a fluorescence baseline for 2–3 s (2–3 images at 1 Hz; excitation, 480 ± 20 nm; emission, 540 ± 30 nm), 20 μL (2×) of test compound or vehicle was added to the cells, and the response was measured. 140 s later, 10 μL (5X) of an EC_20_ concentration of ACh or vehicle was added to the cells, and the response of the cells was measured. Approximately 125 s later, an EC_80 or_ EC_Max_concentration of ACh was added. Calcium fluorescence was recorded as fold over basal fluorescence and raw data were normalized to the maximal response to ACh. Compound-evoked decreases in calcium response in the presence of ACh EC_80_ agonist were determined as inhibition activity, and potency (IC_50_) and maximum inhibition responses (% ACh_Min_) of compounds were determined using a four-parameter logistical equation using GraphPad Prism (La Jolla, CA) or the Dotmatics software platform (Woburn, MA)

$$y=\mathrm{bottom}+\frac{\mathrm{top}-\mathrm{bottom}}{1+10^{\left(\mathrm{logEC}50-A\right)\mathrm{Hillslope}}}$$

where *A* is the molar concentration of the compound; bottom and top denote the lower and upper plateaus of the concentration–response curve; HillSlope is the Hill coefficient that describes the steepness of the curve; and EC_50_ is the molar concentration of compound required to generate a response halfway between the *t*op and bottom.

## Supplementary Material



## Notes

General methods for the synthesis and characterization of key compounds, experimental details for calcium mobilization assays, *in vitro* and *in vivo* DMPK protocols, soft spot analysis protocols, and adult rat DRG sensory neuron culture protocols (PDF)

## Abbreviations

AC: Adenylate cyclase
ACh: acetylcholine
AMPK: AMP-activated protein kinase
CLhep: hepatic clearance
CNS: central nervous system
DMAP: 4-dimethylaminopyridine
DRG: dorsal root ganglia
DMPK: drug metabolism and pharmacokinetics
ER: efflux ratio
GPCRs: G protein-coupled receptors
hM1: muscarinic acetylcholine receptor subtype 1, human
hM2: muscarinic acetylcholine receptor subtype 2, human
K_B_: binding affinity
mAChR: muscarinic acetylcholine receptor
nAChR: nicotinic acetylcholine receptor
MDGF: medium dose cocktail of growth factors
MLPCN: Molecular Libraries Screening Center Network
PK: pharmacokinetic
PNS: peripheral nervous system
rM1: muscarinic acetylcholine receptor subtype 1, rat
RRMS: relapsing-remitting multiple sclerosis
SAR: structure–activity relationship
