# Correction to “Discovery of VU6077564: A Selective M_1_ Antagonist that Promotes Neurite Outgrowth in Adult Sensory Neurons”

**DOI:** 10.1021/acschemneuro.6c00717

**Published:** 2026-09-29

**Authors:** Tomayo I. Berida, Cayden J. Dodd, Joshua C. Wilkinson, Darrell R. Smith, Sichen Chang, Christopher C. Presley, Li Peng, Snehal Sant, Srinivasan Krishnan, Irene Zagol-Ikapitte, Katherine J. Watson, Analisa Thompson Gray, Alice L. Rodriguez, Hyekyung P. Cho, Carrie K. Jones, Olivier Boutaud, Colleen M. Niswender, Darren W. Engers, Paul Fernyhough, Craig W. Lindsley, Elizabeth S. Childress

**Affiliations:** † Warren Center for Neuroscience Drug Discovery, 5718 Vanderbilt University, Nashville, Tennessee 37232, United States; ‡ Department of Pharmacology, 12327 Vanderbilt University School of Medicine, Nashville, Tennessee 37232, United States; § Vanderbilt Institute for Therapeutic Advances, Vanderbilt University, Nashville, Tennessee 37232, United States; ∥ Division of Neurodegenerative & Neurodevelopmental Disorders, St. Boniface Hospital Albrechtsen Research Centre, 8664 University of Manitoba, Winnipeg R3T 2N2, Canada; ⊥ Vanderbilt Brain Institute, Vanderbilt University School of Medicine, Nashville, Tennessee 37232, United States; # Vanderbilt Kennedy Center, Vanderbilt University School of Medicine, Nashville, Tennessee 37232, United States; ⊗ Department of Pharmacology & Therapeutics, Max Rady College of Medicine, Rady Faculty of Health Sciences, University of Manitoba, Winnipeg R3E 0T6, Canada; ◇ Department of Chemistry, Vanderbilt University, Nashville, Tennessee 37232, United States; ◆ Department of Biochemistry, Vanderbilt University, Nashville, Tennessee 37232, United States

Correction 1: In the original article, the rat *in vivo* clearance value for VU6077564 (**31d**) was reported incorrectly. The correct rat *in vivo* clearance value has been provided in Table 7. The interpretation of this result has been corrected from high to moderate. All other results, interpretations, and conclusions in the article are correct.

**Table 7:** Additional *In Vitro* Pharmacology Characterization, *In Vitro* DMPK, and rat *In Vivo* PK Profile of Analogs **28f** and **31d**

	**VU6074545 (28f)**	**VU6077564 (31d)**
MW	508.6	522.6
xLogP	1.78	2.28
tPSA (Å)	119.3	119.3
hM_1_ IC_50_ (nM)	71	31
rM_1_ IC_50_ (nM)	50.9	39.5
hM_1_ K_B_ (nM)	25	30
rM_1_ K_B_ (nM)	22	12
hM_2‑5_ (μM)	>10	>10
**Rat *in vitro* PK parameters**		
rCL_int_ (mL/min/kg)	114	94
rCL_hep_ (mL/min/kg)	43.4	40.1
*f*_u,plasma_ (rat)^a^	0.020	0.019
*f*_u,brain_ (rat)^a^	0.036	0.031
MDCKII-MDR1 P-gp ER	48.0	46.6
**Rat *in vivo* PK parameters**		
CL_p_ (mL/min/kg)^b^	17.6	29.4
*V*_ss_ (L/kg)	0.32	27.6
Elim. *t*_1/2_ (h)	0.33	35.2
*K*_p_ ^c^	0.03	0.08
*K*_p,uu_ ^d^	0.05	0.13

a *f*_u_ = Fraction unbound; equilibrium dialysis assay; brain = rat brain homogenates.

b Male Sprague–Dawley rats (*n* = 2); IV PK: 0.2 mg/kg, vehicle = 12% ethanol, 48% PEG400, 40% DMSO (0.5 mL/kg).

c *K*_p_ = total brain to total plasma ratio.

d *K*_p,uu_ = unbound brain (brain *f*_u_ × total brain) to unbound plasma (plasma *f*_u_ × total plasma) ratio.

Correction 2: Page 2746, third paragraph, first sentence.

Compound **31d** displayed moderate *in vivo* clearance in rat (CL_p_ = 29.4 mL/min/kg) with a long elimination half-life of 35.2 h and high volume of distribution (27.6 L/kg).

Correction 3: Page 2746, fourth paragraph, first sentence.

While **31d** displayed moderate *in vivo* clearance in rat, we were still encouraged by its excellent potency and selectivity for M_1_.

Correction 4: Page 2747, Conclusion, second sentence.

VU6077564 displays moderate *in vivo* clearance in rat and is peripherally restricted (*K*_p_ = 0.08, *K*_p,uu_ = 0.13, ER = 46.6).

