# Supplementary material for: Discovery of VU6077564: A Selective M1 Antagonist That Promotes Neurite Outgrowth in Adult Sensory Neurons
Source: ACS Chem Neurosci. 2026 Jul 7;17(14):2739–51. doi: 10.1021/acschemneuro.6c00367 (PMC13377608; doi:10.1021/acschemneuro.6c00367)
Supplement: Supplementary file 1 [file cn6c00367_si_001.pdf]

Supporting Information

Discovery of VU6077564: A Selective M_1 Antagonist that Promotes Neurite Outgrowth in Adult Sensory Neurons

Tomayo I. Berida[a,b], Cayden J. Dodd[a,b], Joshua C. Wilkinson[a,b,c], Darrell R. Smith[d], Sichen Chang[a,b,c], Christopher C. Presley[a,b,c], Li Peng[a,b], Snehal Sant[a,b,c], Srinivasan, Krishnan[a,b,c], Irene Zagol-Ikapitte[a,b,c], Katherine J. Watson[a,b], Analisa Thompson Gray[a,b,c], Alice L. Rodriguez[a,b,c], Hyekyung P. Cho[a,b,c] Carrie K. Jones[a,b,c,e], Olivier Boutaud[a,b,c], Colleen M. Niswender[a,b,c,e,f], Darren W. Engers[a,b,c], Paul Fernyhough[d,g], Craig W. Lindsley[a,b,c,h,i], Elizabeth S. Childress[a,b,c]*

[a]Warren Center for Neuroscience Drug Discovery, Vanderbilt University, Nashville, Tennessee 37232, USA
[b]Department of Pharmacology, Vanderbilt University School of Medicine, Nashville, Tennessee 37232, USA
[c]Vanderbilt Institute for Therapeutic Advances, Vanderbilt University, Nashville, TN 37232, USA
[d]Division of Neurodegenerative & Neurodevelopmental Disorders, St. Boniface Hospital Albrechtsen Research Centre, University of Manitoba, Winnipeg, Canada
[e]Vanderbilt Brain Institute, Vanderbilt University School of Medicine, Nashville, TN 37232, USA
[f]Vanderbilt Kennedy Center, Vanderbilt University School of Medicine, Nashville, TN 37232, USA
[g]Department of Pharmacology & Therapeutics, Max Rady College of Medicine, Rady Faculty of Health Sciences, University of Manitoba, Winnipeg, Canada
[h]Department of Chemistry, Vanderbilt University, Nashville, Tennessee 37232, USA
[i]Department of Biochemistry, Vanderbilt University, Nashville, TN 37232, USA

*Corresponding authors' email:
elizabeth.s.childress@vanderbilt.edu

Table of Contents

Experimental Synthetic Procedures and Spectroscopic Data 2
General Synthetic Methods 2
General Instrumentation Methods. 2
General Procedure for the Preparation of Analogs **28f** (Scheme 1D) and **31d** (Scheme 2). 4
Molecular Pharmacology Methods 8
Calcium Mobilization Assay 8
Measurement of Myo-Inositol 1 phosphate (IP1) accumulation in Homogeneous Time-Resolved Fluorescence (HTRF) Assay 10
Figure S1. 11
Figure S2. 12
DMPK Methods 12
IV PK and plasma-brain level determination (PBL). 12
Binding in plasma from rat 13
Binding in brain homogenate from rat 14
Intrinsic clearance in rat liver microsomes 14
LC-MS/MS Analysis 15

MDCKII-MDR1 P-gp efflux ratio 16
Soft Spot Analysis Methods 17
Instrument setup 17
Adult Rat DRG Sensory Neuron Culture Methods 18
Assessment of total neurite outgrowth 18
Figure S3. 19
References 19

Experimental Synthetic Procedures and Spectroscopic Data

General Synthetic Methods.

All reactions were carried out employing standard chemical techniques. Solvents used for extraction, washing, and chromatography were HPLC grade. All reagents were purchased from commercial sources and were used without further purification.

Automated flash column chromatography was performed on a Biotage Isolera 1 or a Teledyne ISCO CombiFlash system. RP-HPLC was performed on a Gilson preparative reversed-phase HPLC system comprised of a 333 aqueous pump with solvent-selection valve, 334 organic pump, GX-271 or GX-281 liquid hander, two column switching valves, and a 155 UV detector. Absorbance was typically monitored at 215 or 220 nm. Column: Phenomenex Axia-packed Gemini C18, 5 µm. Mobile phase: CH_3CN in H_2O (0.1% TFA) or CH_3CN in H_2O (0.05% *v/v* NH_4OH) under the specified gradient, then hold 95% CH_3CN in 5% aqueous phase, 50 mL/min, 23° C. All compounds were found to be >95% pure by LCMS analysis.

***Safety statement**: no unexpected or unusually high safety hazards were encountered.*

General Instrumentation Methods.

All NMR spectra were recorded on a 400 MHz AMX Bruker NMR spectrometer. ^{1}H and ^{13}C chemical shifts are reported in δ values in ppm downfield with the deuterated solvent as the internal standard. Data are reported as follows: chemical shift, multiplicity (s = singlet, d = doublet, t = triplet, q = quartet, b = broad, m = multiplet), integration, coupling constant (Hz).

Low resolution mass spectra (LRMS) were obtained on an Agilent 6120/6150 or Waters QDa (Performance) SQ MS with ESI source. *Method A (Agilent 6120/6150):* MS parameters were as follows: fragmentor: 70, capillary voltage: 3000 V, nebulizer pressure: 30 psig, drying gas flow: 13 L/min, drying gas temperature: 350 ºC. Samples were introduced via an Agilent 1290 UHPLC comprised of a G4220A binary pump, G4226A ALS, G1316C TCC, and G4212A DAD with ULD flow cell. UV absorption was

generally observed at 215 nm and 254 nm with a 4 nm bandwidth. Column: Waters Acquity BEH C18, 1.0 x 50 mm, 1.7 um. Gradient conditions: 5% to 95% CH_3CN in H_2O (0.1% TFA) over 1.4 min, hold at 95% CH_3CN for 0.1 min, 0.5 mL/min, 55 ºC. *Method B (Agilent 6120/6150):* MS parameters were as follows: fragmentor: 100, capillary voltage: 3000 V, nebulizer pressure: 40 psig, drying gas flow: 11 L/min, drying gas temperature: 350 ºC. Samples were introduced via an Agilent 1200 HPLC comprised of a degasser, G1312A binary pump, G1367B HP-ALS, G1316A TCC, G1315D DAD, and a Varian 380 ELSD (if applicable). UV absorption was generally observed at 215 nm and 254 nm with a 4 nm bandwidth. Column: Thermo Accucore C18, 2.1 x 30 mm, 2.6 um. Gradient conditions: 7% to 95% CH_3CN in H_2O (0.1% TFA) over 1.6 min, hold at 95% CH_3CN for 0.35 min, 1.5 mL/min, 45 ºC. *Method C (Waters QDa (Performance) SQ MS)*: MS parameters were as follows: cone voltage: 15 V, capillary voltage: 0.8 kV, probe temperature: 600° C. Samples were introduced via an Acquity I-Class PLUS UPLC comprised of a BSM, FL-SM, CH-A, and PDA. UV absorption was generally observed at 215 nm and 254 nm; 4 nm bandwidth. Column: Phenomenex EVO C18, 1.0 x 50 mm, 1.7 um. Column temperature: 55 °C. Flow rate: 0.4 mL/min. Default gradient: 5% to 95% CH_3CN (0.05% TFA) in H_2O (0.05% TFA) over 1.4 min (curve 6), hold at 95% CH_3CN for 0.1 min. "Polar" (2% to 70% CH_3CN (0.05% TFA) in H_2O (0.05% TFA) over 0.8 min (curve 6), transition to 95% CH_3CN over 0.1 min (curve 6), hold at 95% CH_3CN for 0.6 min.) and "Non-Polar" (40% to 95% CH_3CN (0.05% TFA) in H_2O (0.05% TFA) over 1.4 min (curve 6), hold at 95% CH_3CN for 0.1 min.) gradients were also available. *Method D (Waters QDa (Performance) SQ MS):* MS parameters were as follows: cone voltage: 15 V, capillary voltage: 0.8 kV, probe temperature: 600 °C. Samples were introduced via an Acquity I-Class PLUS UPLC comprised of a BSM, FL-SM, CH-A, and PDA. UV absorption was generally observed at 215 nm and 254 nm with a 4 nm bandwidth. Column: Phenomenex EVO C18, 1.0 x 50 mm, 1.7 um. Column temperature: 55 °C. Flow rate: 0.4 mL/min. Default gradient: 5% to 95% CH_3CN in H_2O (5 mM NH_4HCO_3) over 1.4 min (curve 6), hold at 95% CH_3CN for 0.1 min. "Polar" (2% to 70% CH_3CN in H_2O (5 mM NH_4HCO_3) over 0.8 min (curve 6), transition to 95% CH_3CN over 0.1 min (curve 6), hold at 95% CH_3CN for 0.6 min.) and "Non-Polar" (40% to 95% CH_3CN in H_2O (5 mM NH_4HCO_3) over 1.4 min (curve 6), hold at 95% CH_3CN for 0.1 min.) gradients were also available.

High resolution mass spectra (HRMS) were obtained on an Agilent 6540 UHD Q-TOF with ESI source. MS parameters were as follows: fragmentor: 150, capillary voltage: 3500 V, nebulizer pressure: 60 psig, drying gas flow: 13 L/min, drying gas temperature: 275 ºC. Samples were introduced via an Agilent 1200 UHPLC comprised of a G4220A binary pump, G4226A 3 ALS, G1316C TCC, and G4212A DAD with ULD flow cell. UV absorption was observed at 215 nm and 254 nm with a 4 nm bandwidth. Column: Agilent Zorbax Extend C18, 1.8 µm, 2.1 x 50 mm. Gradient conditions: 5% to 95% CH_3CN in H_2O (0.1% formic acid) over 1 min, hold at 95% CH_3CN for 0.1 min, 0.5 mL/min, 40 ºC.

General Procedure for the Preparation of Analogs **28f** (Scheme 1D) and **31d** (Scheme 2).

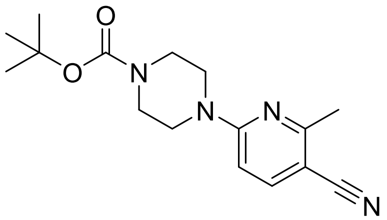

***tert*-butyl 4-(5-cyano-6-methylpyridin-2-yl)piperazine-1-carboxylate**. Step 1: To a solution of 6-chloro-2-methylnicotinonitrile (60 mg, 0.39 mmol) in MeCN (2.6 mL) was added *tert*-butyl piperazine-1-carboxylate (110 mg, 0.59 mmol) and DIPEA (0.137 mL, 0.79 mmol). After stirring at 80 °C for 17 h, the reaction mixture was cooled to rt, diluted with water, and extracted with DCM (3x). The combined organics were passed through a phase separator and concentrated. Purification via normal-phase column chromatography on silica gel (0-50% EtOAc/hexanes) afforded the desired product as a white solid (110 mg, 93% yield). ^1H NMR (400 MHz, CD_3OD) δ 7.66 (d, J = 9.0 Hz, 1H), 6.68 (d, J = 9.0 Hz, 1H), 3.75 – 3.67 (m, 4H), 3.55 – 3.48 (m, 4H), 2.52 (s, 3H), 1.48 (s, 9H). HRMS (Q-TOF, ES+): Calculated for $C_{16}H_{22}N_4O_2$ $(M+H)^+$, 303.1816**;** Observed, 303.1822.

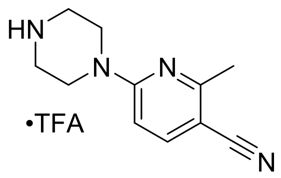

2-methyl-6-(piperazin-1-yl)nicotinonitrile; 2,2,2-trifluoroacetate (**26**). To a solution of *tert*-butyl 4-(5-cyano-6-methylpyridin-2-yl)piperaine-1-carboxylate (100 mg, 0.33 mmol) in DCM (2 mL) was added TFA (0.215 mL, 2.81 mmol). After stirring for 17 h, the mixture was concentrated *in vacuo* and carried forward without further purification in quantitative yield. ^1H NMR (400 MHz, D_2O) δ 7.89 (d, J = 9.1 Hz, 1H), 6.89 (d, J = 9.1 Hz, 1H), 3.99 (dd, J = 5.4, 5.3 Hz, 4H), 3.39 (dd, J = 5.4, 5.3 Hz, 4H), 2.61 (s, 3H). HRMS (Q-TOF, ES+): Calculated for $C_{11}H_{14}N_4$ $(M+H)^+$, 203.1291**;** Observed, 203.1296.

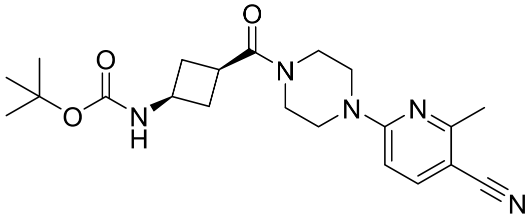

***tert*-butyl ((1*s*,3*s*)-3-(4-(5-cyano-6-methylpyridin-2-yl)piperazine-1-carbonyl)cyclobutyl)carbamate**. To a solution of **26** (107 mg, 0.33 mmol) in DMF (1.4 mL) was added *cis*-3-(*tert*-butyoxycarbonylamino)cyclobutanecarboxylic acid (60 mg, 0.28 mmol), HATU (159 mg, 0.42 mmol), and DIPEA (0.146 mL, 0.84 mmol). After stirring a rt for 17 h, the reaction mixture was syringe filtered and purified using RP-HPLC (20-60% ACN/0.5% aqueous NH_4OH) to give a white solid (40 mg, 36% yield). ^{1}H NMR (400 MHz, CD_3OD) δ 7.68 (d, *J* = 8.9 Hz, 1H), 6.69 (d, *J* = 9.0 Hz, 1H), 4.06 – 3.94 (m, 1H), 3.77 – 3.63 (m, 6H), 3.61 – 3.55 (m, 2H), 3.12 – 3.04 (m, 1H), 2.56 – 2.47 (m, 5H), 2.17 – 2.06 (m, 2H), 1.43 (s, 9H). HRMS: (Q-TOF, ES+): Calculated for $C_{21}H_{29}N_5O_3$ $(M+H)^+$**,** 400.2343**;** Observed, 400.2351.

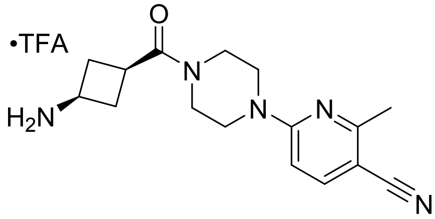

6-(4-((1*s*,3*s*)-3-aminocyclobutane-1-carbonyl)piperazin-1-yl)-2-methylnicotinonitrile; 2,2,2-trifluoroacetate (**27**). To a solution of *tert*-butyl ((1*s*,3*s*)-3-(4-(5-cyano-6-methylpyridin-2-yl)piperazine-1-carbonyl)cyclobutyl)carbamate (37 mg, 0.092 mmol) in DCM (0.5 mL) was added TFA (0.06 mL, 0.7835 mmol). After stirring at rt for 3 h, the mixture was concentrated *in vacuo* and carried forward without further purification in quantitative yield. ^{1}H NMR (400 MHz, D_2O) δ 8.02 (d, *J* = 9.5 Hz, 1H), 7.13 (d, *J* = 9.6 Hz, 1H), 3.93 – 3.73 (m, 9H), 3.45 – 3.31 (m, 1H), 2.73 (s, 3H), 2.70 – 2.61 (m, 2H), 2.44 – 2.32 (m, 2H). HRMS: (Q-TOF, ES+): Calculated for $C_{16}H_{21}N_5O$ $(M+H)^+$**,** 300.1819**;** Observed, 300.1827.

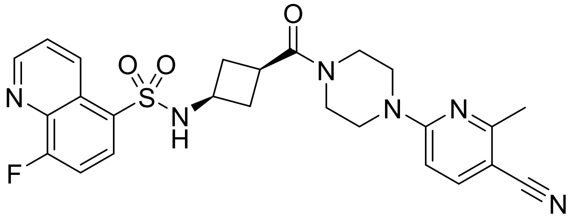

***N*-((1*s*,3*s*)-3-(4-(5-cyano-6-methylpyridin-2-yl)piperazine-1-carbonyl)cyclobutyl)-8-fluoroquinoline-5-sulfonamide** (**28f**). To a solution of **27** (38 mg, 0.092 mmol) in DCM (0.5 mL) was added 8-fluoroquinoline-5-sulfonyl chloride (25 mg, 0.10 mmol) and TEA (0.04 mL, 0.58 mmol). After stirring at rt for 1.5 h, the reaction mixture was diluted with water and extracted with DCM (3x). The combined organics were passed through a phase separator and concentrated. Purification via RP-HPLC (20-60% ACN/0.5% aqueous NH_4OH) afforded an off-white solid (28 mg, 60% yield). ^1H NMR (400 MHz, $(CD_3)_2CO$) δ 9.15 (ddd, J = 8.8, 1.6 Hz, J_{HF} = 1.7 Hz, 1H), 9.07 (dd, J = 4.1, 1.6 Hz, 1H), 8.32 (dd, J = 8.3 Hz, J_{HF} = 5.0 Hz, 1H), 7.79 (dd, J = 8.8, 4.1 Hz, 1H), 7.68 (d, J = 8.9 Hz, 1H), 7.67 (dd, J = 8.3Hz, J_{HF} = 9.9 Hz, 1H), 7.32 (d, J = 9.1 Hz, 1H), 6.69 (d, J = 9.0 Hz, 1H), 3.86 – 3.73 (m, 1H), 3.69 – 3.59 (m, 4H), 3.58 – 3.53 (m, 2H), 3.49 – 3.42 (m, 2H), 2.96 (tt, J = 9.8, 7.9 Hz, 2H), 2.48 (s, 3H), 2.26 – 2.13 (m, 1H), 2.03 – 1.93 (m, 2H). ^{13}C NMR (101 MHz, Acetone) δ 172.11, 161.92, 161.87 (d, J_{CF} = 263.3 Hz), 159.84, 152.32 (d, J_{CF} = 2.1 Hz), 141.74, 139.73 (d, J_{CF} = 11.7 Hz), 134.26 (d, J_{CF} = 2.5 Hz), 134.09 (d, J_{CF} = 4.9 Hz), 131.04 (d, J_{CF} = 9.5 Hz), 126.79 (d, J_{CF} = 2.5 Hz), 124.58, 119.19, 113.08 (d, J_{CF} = 20.1 Hz), 104.73, 96.39, 45.22, 45.13, 44.87, 41.97, 34.79, 34.76, 23.84. HRMS (Q-TOF, ES+): Calculated for $C_{25}H_{25}FN_6O_3S$ $(M+H)^+$, 509.1766**;** Observed, 509.1772.

General Procedure for the Preparation of Analogs **31** (Scheme 2).

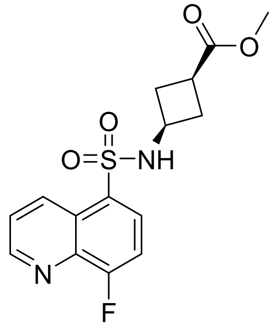

methyl (1*s*,3*s*)-3-((8-fluoroquinoline)-5-sulfonamido)cyclobutane-1-carboxylate. To a solution of *cis*-methyl 3-aminocyclobutanecarboxylate hydrochloride (371 mg, 2.24 mmol) and DIPEA (1.8 mL, 10.18 mmol) in DCM (10 mL) in an ice bath was added 8-fluoroquinoline-5-sulfonyl chloride (500 mg, 2.04 mmol). The resulting reaction mixture was allowed to warm to rt and stirred for 17 h. The reaction was then quenched with saturated aqueous $NaHCO_3$ solution and extracted with DCM (2x). The combined organic layers were concentrated and purified by using normal-phase column chromatography on silica gel (10-100% EtOAc/hexanes) to provide *cis*-methyl 3-[(8-fluoroquinolin-5-yl)sulfonylamino]cyclobutane-1-carboxylate (440 mg, 64% yield) as a white solid. ^1H NMR (400 MHz, CD_3OD) δ 9.18 (ddd, J = 8.8, 1.6 Hz, J_{HF} = 1.6 Hz, 1H), 9.03 (dd, J = 4.3, 1.6 Hz, 1H), 8.31 (dd, J = 8.3 Hz, J_{HF} = 5.0 Hz, 1H), 7.82 (dd, J = 8.9, 4.3 Hz, 1H), 7.63 (dd, J = 8.3 Hz, J_{HF} = 9.9 Hz, 1H), 3.76 – 3.61 (m, 1H), 3.57 (s, 3H), 2.67 (tt, J =

10.0, 7.9 Hz, 1H), 2.22 – 2.14 (m, 2H), 1.96 – 1.86 (m, 2H). HRMS: (Q-TOF, ES+): Calculated for $C_{15}H_{15}FN_2O_4S$ $(M+H)^+$**,** 339.0809**;** Observed, 339.0814.

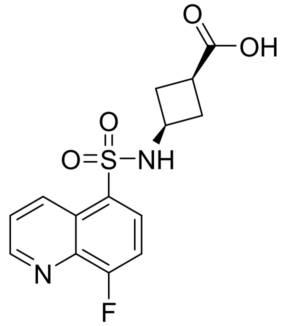

(1*s*,3*s*)-3-((8-fluoroquinoline)-5-sulfonamido)cyclobutane-1-carboxylic acid (**30**). To *cis*-methyl 3-[(8-fluoroquinolin-5-yl)sulfonylamino]cyclobutane-1-carboxylate (170 mg, 0.5 mmol) was added water (1 mL) and TFA (1 mL, 13.06 mmol), and the mixture was stirred at 100 °C for 2 h. The solvent was removed *in vacuo* to afford a white powder that was carried forward without further purification (162 mg). ^{1}H NMR (400 MHz, CD_3OD) δ 9.18 (ddd, J = 8.8, 1.6 Hz, J_{HF} = 1.6 Hz, 1H), 9.03 (dd, J = 4.2, 1.5 Hz, 1H), 8.31 (dd, J = 8.3 Hz, J_{HF} = 4.9 Hz, 1H), 7.82 (dd, J = 8.9, 4.2 Hz, 1H), 7.63 (dd, J = 8.3 Hz, J_{HF} = 9.9 Hz, 1H), 3.68 (tt, J = 9.2, 7.5 Hz, 1H), 2.62 (tt, J = 10.0, 7.9 Hz, 1H), 2.32 – 2.06 (m, 2H), 1.97 – 1.84 (m, 2H). HRMS: (Q-TOF, ES+): Calculated for $C_{14}H_{13}FN_2O_4S$, $(M+H)^+$, 325.0653**;** Observed, 325.0656.

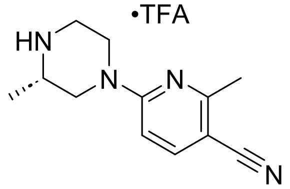

(*S*)-2-methyl-6-(3-methylpiperazin-1-yl)nicotinonitrile; (2,2,2-trifluoroacetate). *Tert*-butyl (2*S*)-2-methyl-1-piperazinecarboxylate (74 mg, 0.37 mmol) was dissolved in MeCN (1 mL). Cesium carbonate (71 mg, 0.73 mmol) and 6-fluoro-2-methylnicotinonitrile (50 mg, 0.37 mmol) were added sequentially, and the reaction mixture was refluxed for 17 h. The reaction mixture was diluted with water followed by extraction with DCM (2x). The combined organic layers were passed through a phase separator and concentrated. To the concentrated residue was added DCM (0.5 mL) and TFA (0.113 mL, 1.47 mmol). After stirring at rt for 2 h, solvents were removed *in vacuo*, and the sample carried forward without further purification as a brown solid (120.9 mg). ^{1}H NMR (400 MHz, CD_3OD) δ 7.76 (d, J = 8.9 Hz, 1H), 6.82 (d, J = 8.9 Hz, 1H), 4.66–4.55 (m, 2H), 3.48 (dt, J = 12.6, 2.8 Hz, 1H), 3.44–3.35 (m, 1H), 3.34–3.25 (m, 1H), 3.18 (td, J = 12.2, 3.3 Hz, 1H), 3.07 (dd, J = 14.4, 10.6 Hz, 1H), 2.55 (s, 3H), 1.39 (d, J = 6.6 Hz, 3H). HRMS (Q-TOF, ES+): Calculated for $C_{12}H_{16}N_4$ $(M+H)^+$, 217.1448**;** Observed, 217.1448.

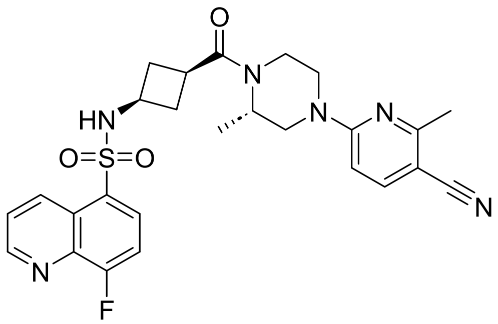

***N*-((1*R*,3*s*)-3-((*S*)-4-(5-cyano-6-methylpyridin-2-yl)-2-methylpiperazine-1-carbonyl)cyclobutyl)-8-fluoroquinoline-5-sulfonamide** (**31d**). To a mixture of intermediate **30** (20 mg, 0.062 mmol), (*S*)-2-methyl-6-(3-methylpiperazin-1-yl)nicotinonitrile; (2,2,2-trifluoroacetate) (20 mg, 0.062 mmol), and HATU (28 mg, 0.074 mmol) in DMF (1 mL) was added DIPEA (0.035 mL, 0.25 mmol). After stirring at rt for 2 h, the mixture was syringe filtered and purified using RP-HPLC (15-70% ACN/0.5% aqueous NH_4OH) to give a white solid (15 mg, 47% yield). ^{1}H NMR (400 MHz, $CDCl_3$) (mixture of rotamers) δ 9.13–9.02 (m, 2H), 8.28 (dd, J = 8.3 Hz, J_{HF} = 4.9 Hz, 1H), 7.63 (dd, J = 8.7, 4.2 Hz, 1H), 7.58 (d, J = 8.9 Hz, 1H), 7.46 (dd, J = 8.3 Hz, J_{HF} = 9.4 Hz, 1H), 6.40 (d, J = 8.9 Hz, 1H), 6.20 (d, J = 9.6 Hz, 0.5H), 6.11 (d, J = 9.4 Hz, 0.5H), 4.84–4.72 (m, 0.5H), 4.48–4.34 (m, 1H), 4.24 (d, J = 13.4 Hz, 0.5H), 4.14 (d, J = 12.1 Hz, 0.5H), 4.06–3.90 (m, 1H), 3.79 (h, J = 8.2 Hz, 1H), 3.55 – 3.31 (m, 1H), 3.31–3.22 (m, 1H), 3.16–2.93 (m, 1.5H), 2.86 (dt, J = 17.7, 8.4 Hz, 1H), 2.57 (s, 3H), 2.39 – 2.26 (m, 2H), 2.19–2.00 (m, 2H), 1.13 (dd, J = 20.2, 6.7 Hz, 3H). ^{13}C NMR (101 MHz, $CDCl_3$) (mixture of rotamers) δ 172.2 (d, J_{CF} = 39.6 Hz), 161.8, 161.4 (d, J_{CF} = 265.5 Hz), 158.8, 151.4, 141.2, 138.9 (d, J_{CF} = 12.0 Hz), 133.5, 132.1 (d, J_{CF} = 3.3 Hz), 130.3 (d, J_{CF} = 9.5 Hz), 126.1 (d, J_{CF} = 2.8 Hz), 123.8, 118.7, 112.2 (d, J_{CF} = 20.1 Hz), 103.2 (d, J_{CF} = 7.1 Hz), 96.3, 49.5, 48.3, 48.0, 46.1, 44.9, 44.7, 44.4, 40.4, 36.8, 34.8, 34.5, 34.4, 34.2, 30.7, 30.2, 23.7, 17.6, 16.0. HRMS (Q-TOF, ES+): Calculated for $C_{26}H_{27}FN_5O_3S$ $(M+Na)^+$, 545.1742**;** Observed, 545.1741

Molecular Pharmacology Methods

Calcium Mobilization Assay

Compound-evoked decrease to an EC_{80} concentration of acetylcholine (ACh) in intracellular calcium were measured using Chinese hamster ovary (CHO) cells stably expressing human muscarinic receptors (M_1–M_5; M_2 and M_4 cells were co-expressed with chimeric G_{qi5}) or rat M_1. The stable cells were cultured in F12 medium containing 10% fetal bovine serum, 20 mM HEPES, 100 units/mL antibiotics/antimycotic, 0.5 mg/mL G418, and 0.2 mg/mL hygromycin (M_2 and M_4 G_{qi5} co-expressing cells only). All reagents used were from Life Technologies (Carlsbad, CA) unless otherwise noted.

Briefly, the day before the assay, cells (15,000 cells/20 μL/well) were plated in black-walled, clear-bottomed, 384 well plates (Greiner Bio-One, Monroe, NC) in the culture medium without G418 and hygromycin and then incubated overnight at 37 °C in the presence of 5% CO_2. The next day, calcium assay buffer (Hank's balanced salt solution (HBSS), 20 mM HEPES, 2.5 mM Probenecid, 4.16 mM sodium bicarbonate (Sigma-Aldrich, St. Louis, MO)) was prepared to dilute compounds, agonists, and Fluo-4-acetomethoxyester (Fluo-4-AM, Ion Biosciences), fluorescent calcium indicator dye. Compounds were serially diluted 1:3 or 1:5 into 10-point concentration response curves (CRC) in DMSO using the Bravo liquid handler (Agilent, Santa Clara, CA), transferred to a 384 well daughter plates using an Echo acoustic liquid handler (Beckman Coulter, Indianapolis, Indiana), and diluted in assay buffer to a 2X final concentration. The agonist plates were prepared using acetylcholine (ACh, Sigma-Aldrich, St. Louis, MO) concentrations for the EC_{20}, $EC_{80,}$ and EC_{Max} responses by diluting in assay buffer to a 5X final concentration. The 2X dye solution (2.3 μM) was prepared by mixing a 2.3 mM Fluo-4-AM stock in DMSO with 10% (w/v) pluronic acid F-127 in a 1:1 ratio in assay buffer. Using a microplate washer (BioTek, Winooski, VT), cells were washed with assay buffer 3 times to remove medium. After the final wash, 20 μL of assay buffer remained in the cell plates. Immediately, 20 μL of the 2X dye solution (final 1.15 μM) was added to each well of the cell plate using a Multidrop Combi dispenser (Thermo Fisher, Waltham, MA). After cells were incubated with the dye solutions for 45 min at 37 °C in the presence of 5% CO_2, the dye solutions were removed and replaced with assay buffer using a microplate washer, leaving 20 μL of assay buffer in the cell plate, and the cell plate allowed to incubate for 10 min at 37 °C. The compound, agonist, and cell plates were placed inside the Functional Drug Screening System (FDSS 7000 or μCell kinetic imaging plate reader, Hamamatsu, Japan) to measure the calcium flux. After establishment of a fluorescence baseline for 2-3 seconds (2-3 images at 1 Hz; (excitation, 480 nm; emission, 530 nm), 20 μL (2X) of test compound or vehicle was added to the cells, and the response was measured. 140 seconds later, 10 μL (5X) of an EC_{20} concentration of ACh or vehicle was added to the cells, and the response of the cells was measured. Approximately 125 seconds later, an EC_{80} or EC_{Max} concentration of ACh was added. Calcium fluorescence was recorded as fold over basal fluorescence and raw data were normalized to the maximal response to ACh. Compound-evoked decreases in calcium response in the presence of ACh EC_{80} agonist were determined as antagonist activity, and potency (IC_{50}) and maximum inhibition responses (% ACh_{Min}) of compounds were determined using a four-parameter logistical equation using GraphPad Prism (La Jolla, CA) or the Dotmatics software platform (Woburn, MA)

$$y = bottom + \frac{top - bottom}{1 + 10^{(LogEC50 - A)Hillslope}}$$

where *A* is the molar concentration of the compound; *bottom* and *top* denote the lower and upper plateaus of the concentration-response curve; HillSlope is the Hill coefficient that describes the steepness of the

curve; and EC_{50} is the molar concentration of compound required to generate a response halfway between the *top* and *bottom*.

Measurement of Myo-Inositol 1 phosphate (IP1) accumulation in Homogeneous Time-Resolved Fluorescence (HTRF) Assay

Competitive antagonist mode of action by compounds was examined in progressive fold-shift experiments where acetylcholine-induced IP1 accumulation in human or rat M_1-CHO cells was measured in the absence and presence of increasing concentration of compounds using HTRF IP-One Gq Detection Kit (Revvity, 62IPAPEC). On the day of assay, test compounds (2 mM stock) were serially diluted 1:3 into 11-point concentration response curves in DMSO and 25 nl of the diluted compounds or DMSO were transferred to each well of a white-wall cell assay plate (Griener #781080) using Echo acoustic liquid handler. Acetylcholine agonist plate was prepared in a 384 well plate (Greiner, #781281) by serial diluting 1:3 into 9-point CRC in IP1 stimulation buffer (Revvity, #62IP1FDG) to a 3X final concentration. The human or rat M_1-CHO cells were detached from the cell culture dish using enzyme free dissociation buffer (Gibco, #13151-014) and resuspended in IP1stimulation buffer. The cell suspension (20,000 cells / 10 µl/ well) was directly added into the cell plate containing the compound CRC or DMSO using Assist Plus liquid handler (Integra, Hudson, NH). Immediately, the cell plate was placed in a humidified incubator (37 °C in the presence of 5% CO_2) for 20 minutes to equilibrate the antagonist-receptor complex. After 20 min incubation, the diluted agonist CRC or assay buffer (5 µl / well) from the prepared agonist plate was quickly added to the cell plate by stamping using the 384 well Bravo liquid handler. The cell plate was returned to the incubator for 45 minutes to allow sufficient receptor activation and IP1 accumulation. During the stimulation time, IP1 detection reagents (IP1-d2 acceptor and IP1-terbium cryptate antibody donor) were diluted in lysis and detection buffer (Revvity, #62CL6FDF). Stimulation was stopped by adding 6 µl / well of the detection reagents and the plate was incubated for 1 hour at room temperature. Using an EnVision Plate reader (Perkin Elmer), HTRF signals were measured in two channels, 665 and 620 nm. HTRF emission ratios (665/620) were used for data analysis. Acetylcholine EC_{50} values were determined for each antagonist concentration or DMSO using a four-parameter logistical equation using GraphPad Prism. Using the acetylcholine EC_{50} values, Schild analysis was performed and Dose ratios (DR) were determined to calculate the equilibrium dissociation constant for antagonist (K_B).

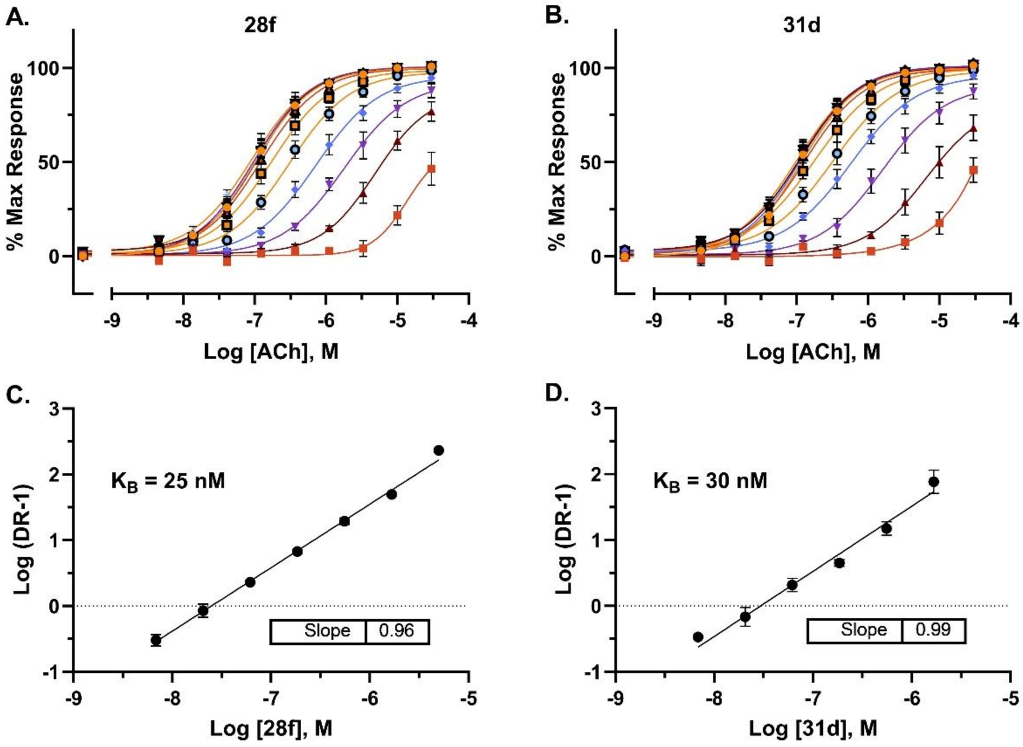

Figure S1. Progressive fold shift and Schild regression analysis of **28f** and **31d** at human M_1. Concentration response curves (CRCs) of ACh-induced IP1 accumulation measured at human M_1-CHO cells incubated with varying concentrations of (A) **28f** and (B) **31d**. Schild analysis of (C) **28f** and (D) **31d**, where the shift in the EC_{50} of ACh due to inhibitor (DR-1) is plotted as a function of inhibitor concentration. CRC data for progressive fold shift were normalized to 30 µM ACh (100%) and no acetylcholine (0%) conditions. Data represent Mean ± SEM, n = 2.

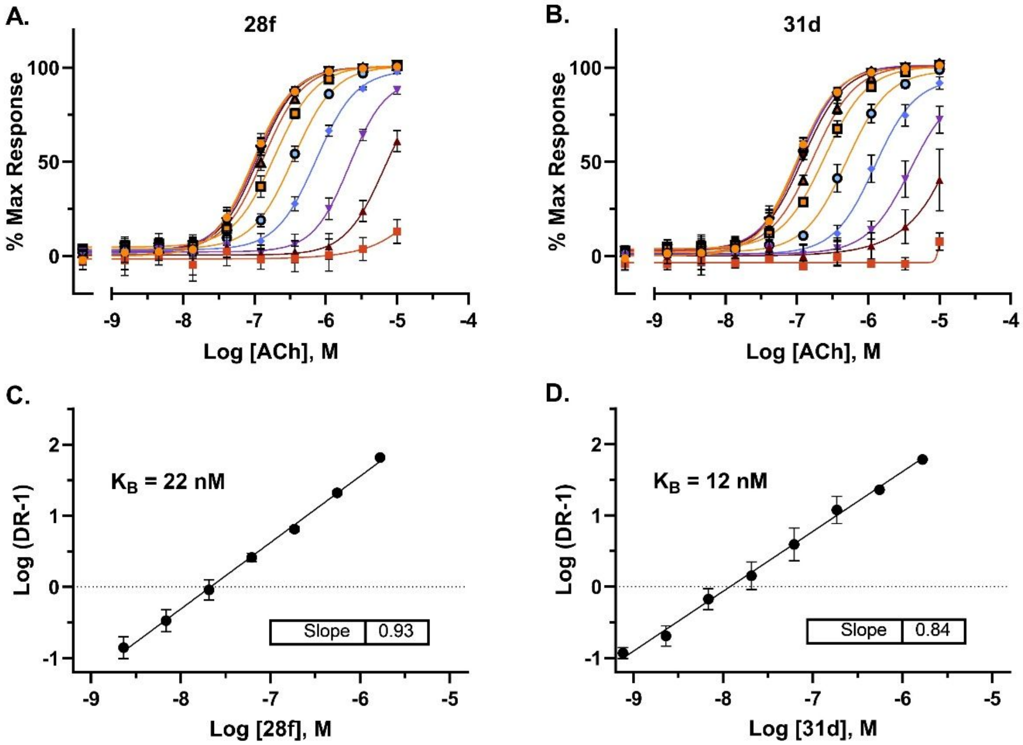

Figure S2. Progressive fold shift and Schild regression analysis of **28f** and **31d** at rat M_1. Concentration response curves (CRCs) of ACh-induced IP1 accumulation measured at rat M_1-CHO cells incubated with varying concentrations of (A) **28f** and (B) **31d**. Schild analysis of (C) **28f** and (D) **31d**, where the shift in the EC_{50} of ACh due to inhibitor (DR-1) is plotted as a function of inhibitor concentration. CRC data for progressive fold shift were normalized to 10 µM ACh (100%) and no acetylcholine (0%) conditions. Data represent Mean ± SEM, n = 2.

<u>DMPK Methods</u>

<u>IV PK and plasma-brain level determination (PBL).</u>

In-life phase

Compounds were formulated as a solution in ethanol, PEG400, and DMSO (1:4:5 v/v, respectively) at a concentration of 1 mg/mL and administered as a single 0.2 mg/kg IV dose (0.5 mL/kg) to male, Sprague Dawley rats (n = 1; 342 gram body weights) via injection into a surgically-implanted jugular vein catheter. Blood samples were collected serially from a surgically implanted carotid artery catheter in each animal over multiple post-administration time points (0.033, 0.117, 0.25, 0.5, 1, 2, 4, 7, and 24 hours) into chilled, K2EDTA anticoagulant-fortified tubes and immediately placed on wet ice. The blood samples were then centrifuged (1700 rcf, 5 minutes, 4 ˚C) in order to obtain plasma samples, which were stored at -80 ˚C until analysis by LC-MS/MS.

For determination of the brain over plasma ratio (K_p), compounds were formulated in 10% ethanol, 40% PEG400 and 50% DMSO (v/v/v) and administered as a single 0.2 mg/kg IV dose (0.5 mL/kg) to male, Sprague Dawley rats (n = 1; 316 gram body weights) via injection into a surgically-implanted jugular vein catheter. At 15 min post dosing, blood sample was collected serially (i.e., terminally) into chilled, K2EDTA anticoagulant-fortified tube and immediately placed on wet ice. The blood sample was then centrifuged (1700 rcf, 5 minutes, 4 ˚C) to obtain plasma sample. At the same post-administration time point, whole brain sample was obtained by rapid dissection, rinsed with saline, and immediately frozen in individual tissue collection box (dry ice). All brain and plasma samples were stored at -80 ˚C until analysis by LC-MS/MS.

Samples preparation for bioanalysis

Plasma samples from the in-life phase of the study were thawed at ambient temperature (benchtop) and then aliquots (20 µL per sample) were transferred to a 96-shallow-well (V-bottom) plate. Matrix-matched quality control (QC) samples and a standard curve of VU6067104 (1 mg/mL DMSO stock solution) were prepared in blank rat plasma (K2EDTA-treated) or blank brain homogenate via serial dilution and transferred (20 µL each) to the plate along with multiple blank plasma and brain homogenate samples. Acetonitrile (120 µL) containing IS (10 nM carbamazepine) was added to each well of the plate to precipitate protein. The plate was then centrifuged (4000 rcf, 5 minutes, ambient temperature) and the resulting supernatants (60 µL each) were transferred to a new 96-shallow-well (V-bottom) plate containing an equal volume (60 µL per well) of water (Milli-Q purified). The plate was then sealed in preparation for LC-MS/MS analysis.

Preparation of brain samples was identical to that of plasma samples except for the following modifications. While thawing, brains were weighed (inside their collection boxes using a universal empty collection box tare weight) and then subjected to mechanical homogenization (Mini-BeadBeater™, BioSpec Products, Inc., Bartlesville, OK) in the presence of zirconia/silica beads (1.0 mm) and extraction buffer (isopropanol:water, 7:3, v/v; 3 mL per sample, corrected for post-quantitation). Homogenized brain samples were then centrifuged (4000 rcf, 5 minutes, ambient temperature), and 5 µL of the supernatant was diluted in 15 µL of blank plasma for quantification of the analyte. The plasma standard curve and QCs were used for compounds quantitation in brain.

Binding in plasma from rat

Determination of compounds' fraction unbound (f_u) in plasma from rat was conducted *in vitro* via equilibrium dialysis using HTDialysis membrane plates. Dialysis membranes (four paired strips per HTD assay) were hydrated as described by the manufacturer and inserted into the HTD plate, which was

assembled and prepared for sample addition by the dispensing of blank buffer (DPBS, 100 µL/well) into the 'top half' of each membrane-split well. Each compound was diluted into plasma from each species (5 µM final concentration), which was aliquoted in triplicate to the 'bottom half' of the prepared HTD plate wells. The HTD plate was sealed and incubated for 6 hours at 37 °C. Following incubation, each well (both top and bottom halves) were transferred (20 µL) to the corresponding wells of a 96-shallow-well (V-bottom) plate. The daughter plates were then matrix-matched (buffer side wells received equal volume of plasma, and plasma side wells received equal volume of buffer), and extraction solution (120 µL; acetonitrile containing 50 nM carbamazepine as IS) was added to all wells of both daughter plates to precipitate protein and extract test article. The plates were then sealed and centrifuged (3500 rcf) for 10 minutes at ambient temperature. Supernatant (60 µL) from each well of the daughter plates was then transferred to the corresponding wells of new daughter plates (96-shallow-well, V bottom) containing water (Milli-Q, 60 µL/well), and the plates were sealed in preparation for LC-MS/MS analysis (see below).

f_u was calculated as (analyte to IS MS peak area ratio from Trans-buffer side) / (analyte to IS MS peak area ratio from Cis-plasma side). Mean values for each species were calculated from 3 replicates.

Binding in brain homogenate from rat

Determination of fraction unbound (f_u) in brain homogenate from rat was conducted using the same methodology and procedure than described for plasma protein binding assay with the following modifications: 1) a final compound concentration of 1 µM was used, 2) naïve rat brains were homogenized in DPBS (1:3 composition of brain: DPBS, w/w) using a Mini-Bead Beater™ machine in order to obtain brain homogenate.

The diluted fraction unbound (f_{u2}) in brain was calculated as (analyte to IS MS peak area ratio from Trans-buffer side) / (analyte to IS MS peak area ratio from Cis-brain homogenate side). Undiluted fraction unbound for the brain was calculated using the following equation:

$$f_u = \frac{1/4}{\left\{\left(\frac{1}{f_{u2}}\right) - 1\right\} + 1/4}$$

Mean values for each species were calculated from 3 replicates.

Intrinsic clearance in rat liver microsomes

The *in vitro* intrinsic clearance (CL_{int}) was investigated in commercially obtained hepatic microsomes from rat using the substrate depletion (i.e., loss-of-parent vs. time, or $t_{1/2}$ method) approach with analyte detection via liquid chromatography-tandem mass spectrometry (LC-MS/MS). For each species, mean %parent remaining values at each time point were calculated from replicates raw data (analyte:IS peak area ratios) and used to determine *in vitro* $t_{1/2}$ and CL_{int}.

Experiments were carried out using a robot-assisted (TECAN model Evo 200). Compound was incubated (1 µM final concentration) in buffer (100 mM potassium phosphate pH 7.4 with 3 mM $MgCl_2$) containing hepatic microsomes (0.5 mg/mL final concentration) from rat, discretely, at 37 °C under constant orbital shaking. After 5 minutes (pre-incubation), reactions were initiated by addition of nicotinamide adenine dinucleotide phosphate (NADPH, 1 mM final concentration). At selected time intervals (0, 3, 7, 15, 25, and 45 minutes) post-addition of NADPH, aliquots (50 µL) were taken and placed into a 96-shallow-well plate containing ice cold acetonitrile (150 µL) with carbamazepine (IS, 50 nM). The plates were then centrifuged (3000 rcf at 4 °C) for 10 minutes. The supernatants were transferred to a new 96-shallow-well daughter plate and diluted (1:1 v/v) with water (Milli-Q filtered). The plates were then sealed in preparation for LC-MS/MS analysis (see below).

Raw LC-MS/MS peak area data generated from the assay samples were used to construct natural log-transformed %parent remaining vs. time plots (using t = 0 minute post-NADPH addition sample data as starting point set to 100%). *In vitro* compound half-life ($t_{1/2}$) values were obtained using the following equation:

$$t_{1/2} = \frac{Ln(2)}{k}$$

Where k is the slope from linear regression analysis of the natural log-transformed data (using means from all replicates at each time point). Resulting $t_{1/2}$ values were then used to calculate hepatic CL_{int} values according to the following equation and with the use of species-specific scale-up factors for liver weight (grams) per total body weight (kg):

$$CL_{int} = \frac{0.693}{in\ vitro\ t_{1/2}}\ x\ \frac{1\ mL\ incubation}{0.5\ mg\ microsomes}\ x\ \frac{45\ mg\ microsomes}{1\ gram\ liver}\ x\ \frac{45^{a}\ gram\ liver}{kg\ body\ wt}$$

[a]Scale-up factors used are 45 (rat) and 20 (human).[4]

Predicted hepatic clearance (CL_{hep}) was calculated using the following equation:

$$CL_{hep} = \frac{Q_h * CL_{int}}{Q_h + CL_{int}}$$

Q_h represents hepatic blood flow (mL/min/kg): 21 for human, 70 for rat, and 90 for mouse.

LC-MS/MS Analysis

Prepared samples were injected (10 µL each) onto an AB Sciex Triple Quad 4500 mass spectrometer system with an Agilent 1260 Infinity II pump and autosampler. Mass spectrometer conditions are described in **Table S1.** Quantitation of compounds was performed via AB Sciex Multiquant software using the raw analyte:IS peak area ratios. The typical detection range was 0.5 ng/mL to ≥ 5,000 ng/mL utilizing a quadratic equation regression with 1/x2 weighting.

Correction for dilution of all brain samples (in extraction buffer and subsequently in blank plasma, as previously described) was performed post-quantitation. The corrections for dilution in extraction buffer employed correction factors specific to each brain weight (not shown).

Table S1. **LC-MS/MS Conditions***

Injection volume	10 µL	
Mobile phase A	0.5% Formic Acid in Water	
Mobile phase B	0.5% Formic Acid in Acetonitrile	
Flowrate	0.5 mL/min	
Gradient	Time	% Mobile Phase B
	0.0	5
	0.2	5
	0.8	95
	1.5	95
	1.7	5
	2.7	Stop
Column	Fortis C18 (50 x 3.0 mm, 3 µm)	
Data collection and analysis software/version	Analyst v. 1.7.1	
Ionization mode	Positive Electrospray	
Curtain gas (psi)	40	
GS1 (psi)	40	
GS2 (psi)	40	
Capillary voltage (V)	5500	
Source TurboIonSpray® temp. (°C)	500	

MDCKII-MDR1 P-gp efflux ratio

Cell Culture

MDCKII-MDR1 cells were cultured in media consisting of Dulbecco's Modified Eagle's Media (low glucose), 25 mM HEPES, 10% fetal bovine serum, 1% non-essential amino acids, 100 units/ml penicillin/streptomycin and 4mM G418 at 37°C, 5% CO_2 and 85% relative humidity. On day 1, MDCKII-MDR1 cells were seeded at a density of 45,000 cells/well onto Corning (Corning, NY) 24-well transwell plate (0.4 µm pore size, 0.33 cm^2 growth area) and place in the cell culture incubators. The assay was performed on day 5. The transwell plates received fresh media change one day before the experiments to prevent cell starvation.

P-glycoprotein Transwell Assay

All transwell assays were performed in HBSS buffer. Transwell assays were performed at 5 µM concentration of compounds. Transporter studies were initiated by adding dosing solutions into donor compartments and measuring appearance of compounds in receiver compartments after 120 min. Before

incubation, donor samples at 0 min were collected. After 120 min of incubation, samples were collected for both donor and receiver chambers. Post-experimental incubation of lucifer yellow (100 μM) was carried out to confirm the integrity of the cell monolayer during incubation of test compounds. Quinidine and propranolol were used as P-gp substrate and high-passive permeability control respectively. All the data for controls were withing the acceptable range. The % recovery for the test compounds was above 80%.

Data Analysis

To determine apparent permeability (P_{app}) the following equation was used:

$$P_{app} = \frac{dQ}{dt} \times \frac{1}{(A \times C_0)}$$

Where dQ/dt is the rate of appearance of the test compounds in the receiver compartment, A is surface area of the membrane (0.33 cm^2), C_0 is the initial concentration (0 min) of the test compounds in the donor compartment.

The ER was calculated by the following equation:

$$Efflux\ Ratio = \frac{P_{app,\ B-A}}{P_{app,\ A-B}}$$

Soft Spot Analysis Methods

Metabolic soft-spot experiments were performed on a 1 mL scale with slow shaking in open 13 x 100 mm borosilicate test tubes with **16b** (VU6053517) by incubation (20 μM) at 37 °C for approximately 1 hour in a solution containing rat S9 fractions (1 mg/mL) in the presence or absence of NADPH (2 mM) in potassium phosphate buffer (0.1 M) with magnesium chloride (3 mM). The reactions were terminated and protein precipitated by the addition of ice-cold acetonitrile, vortexed, chilled for 30 minutes at 4 °C, and then centrifuged at 3500 rcf for 10 minutes. Supernatants were transferred to new 13 x 100 borosilicate test tubes and were concentrated under a stream of nitrogen gas. Resulting residues were reconstituted in acetonitrile/water (1:1 vol/vol, 400 μL) for LC/MS/MS analysis.

Instrument setup

Samples for metabolite identification were analyzed on an Agilent 1290 UHPLC system consisting of a binary pump (G4220A), autosampler (G4226A), thermostatted column compartment (G1316C), and a diode array detector (G4212A) coupled to the diverter valve of an Agilent 6540 UHD accurate-mass Q-TOF equipped with a dual AJS source. One μL injections were made on an Acquity UPLC BEH C_{18}, 50 x 1.0 mm, 1.7 μm column held at 40 °C with a flow rate of 0.3 mL/min consisting of a binary solvent system

composed of Milli-Q water with 5 mM ammonium bicarbonate (A) and acetonitrile (B). Mobile phase was held at 5% B for 0.5 minutes, increased to 12% B over 2.5 minutes, increased to 25% B over 15 minutes, increased to 40% B over 3 minutes, increased to 95% B over 1 minute, and then held at 95% B for another minute. The diverter valve started in the waste position and switched to the Q-TOF after 0.4 minutes and back to waste after 21 minutes. The Q-TOF was operated in positive mode with drying gas flow of 13 L/min at 275 °C, nebulizer at 60 psi, sheath gas flow of 12 L/min at 400 °C, capillary voltage of 4000 V, a nozzle voltage of 500 V, and collision energies of 10, 20, and 30.

Adult Rat DRG Sensory Neuron Culture Methods

As described previously, intact DRG were isolated and dissociated from adult male Sprague-Dawley rats.[2,3] Neurons were cultured in defined Hams F-12 media in the presence of N2 supplement (0.1 mg/ml transferrin, 20 nM progesterone, 100 µM putrescine, 30 nM sodium selenite, 0.1 mg/ml BSA; MilliporeSigma Canada Ltd, Oakville, Ontario, Canada supplied the additives; Wisent Inc, Saint-Jean-Baptiste, Quebec, Canada provided the culture medium), 0.1 nM insulin (MilliporeSigma Canada Ltd, Oakville, Ontario, Canada) and a low-dose cocktail of neurotrophic factors (0.1 ng/ml NGF, 1.0 ng/ml GDNF, and 0.1 ng/ml NT-3; all from Peprotech, Cranbury, NJ, USA).[4] By delivering growth factors, the goal was to mimic the level of neurotrophic support that sensory neurons receive in vivo. The enrichment of neurons was accomplished by centrifuging in a 15% BSA column (Thermo Fisher Scientific, Carlsbad, California, USA).[5] Most of the fibroblasts and Schwann cells were removed during this process, but a minimal number of satellite cells accounted for approximately 5% to 10% of total cells in the final culture (mostly directly associated with sensory neurons). Cells were plated onto poly-D-L-ornithine hydrobromide (MilliporeSigma Canada, Oakville, Ontario, Canada), and laminin-coated 12 mm glass cover slips. In all studies neurons were cultured in the presence of 10 mM D-glucose. The Canadian Committee on Animal Care (CCAC) guidelines were carefully followed for all animal protocols.

Assessment of total neurite outgrowth

Adult rat sensory neurons grown on glass coverslips were fixed with 2% paraformaldehyde in PBS (pH 7.4) for 15 minutes at room temperature and permeabilized with 0.3% Triton X-100 in PBS for 5 minutes (Products from Thermo Fisher Scientific, Carlsbad, California, USA). Neurons were then incubated in blocking buffer (Roche, Basel, Switzerland) diluted with FBS and 1.0 mM PBS (1:1:3) for 1 hour, then rinsed 3 times with PBS. The primary antibody used was against peripherin (Santa Cruz Biotechnology, Dallas, Texas, USA; 1:1000), which is neuron specific. Plates were incubated at 4°C overnight. The following day, the coverslips were incubated with Cy3-conjugated secondary antibodies (Jackson ImmunoResearch Laboratories, West Grove, Pennsylvania, USA) for 1 hour at room temperature and then

mounted and imaged using a Carl Zeiss Axioscope-2 fluorescence microscope equipped with an AxioCam camera. Random images were captured using Axio-Vision4.8 software. Quantification of total neurite outgrowth was performed by measuring the mean pixel area of captured images using ImageJ software and adjusted for the cell body signal. All values were adjusted for neuronal number. The level of total neurite outgrowth in this culture system has been validated to be similar to collateral sprouting in vivo and directly related to arborizing axonal plasticity.[5]

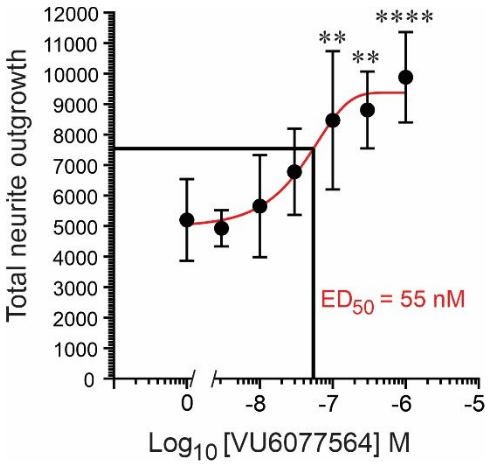

Figure S3. Best line of fit for dose response of **VU6077564** (**31d**) for stimulation of neurite outgrowth and determination of ED_{50}. Semi-log plot of total neurite outgrowth vs concentration of **VU6077564** (**31d**). Values are means +/- SEM, n = 6. Data analyzed using one-way ANOVA with Dunnett's Post hoc test. **p < 0.01 and ****p <0.0001 vs control (zero drug).

References

(1) Kenakin, T. P. The Schild regression in the process of receptor classification. *Can. J. Physiol. Pharmacol.* **1982**, *60*, 249–265.

(2) Fernyhough, P.; Willars, G.B.; Lindsay, R.M.; and Tomlinson, D.R. Insulin and insulin-like growth factor I enhance regeneration in cultured adult rat sensory neurones. *Brain Res*. **1993**, *607*, 117–124.

(3) Mulderry, P.K.; and Lindsay, R.M. Rat dorsal root ganglion neurons in culture express vasoactive intestinal polypeptide (VIP) independently of nerve growth factor. *Neurosci. Lett.* **1990**, *108*, 314-320.

(4) Calcutt, N.A.; Smith, D.R.; Frizzi, K.; Sabbir, M.G.; Chowdhury, S.K.; Mixcoatl-Zecuatl, T.; Saleh, A.; Muttalib, N.; Van der Ploeg, R.; Ochoa, J.; Gopaul, A.; Tessler, L.; Wess, J.; Jolivalt, C. G.; Fernyhough, P. Selective antagonism of muscarinic receptors is neuroprotective in peripheral neuropathy. *J. Clin. Invest.* **2017**, *127*, 608–622.

(5) Gavazzi, I.; Kumar, R.D.; McMahon, S.B.; and Cohen, J. Growth responses of different subpopulations of adult sensory neurons to neurotrophic factors in vitro. *Eur. J. Neurosci.* **1999**, *11*, 3405–3414.

5) Smith, D.S.; and Skene, J.H. A transcription-dependent switch controls competence of adult neurons for distinct modes of axon growth. *J. Neurosci*. **1997**, *17*, 646–658.
